# FGMD: A novel approach for functional gene module detection in cancer

**DOI:** 10.1371/journal.pone.0188900

**Published:** 2017-12-15

**Authors:** Daeyong Jin, Hyunju Lee

**Affiliations:** 1 Korea Environment Institute, Sejong, South Korea; 2 School of Electrical Engineering and Computer Science, Gwangju Institute of Science and Technology, Gwangju, South Korea; Queen’s University Belfast, UNITED KINGDOM

## Abstract

With the increasing availability of multi-dimensional biological datasets for the same samples (i.e., gene expression, microRNAs, copy numbers, mutations, methylations), it has now become possible to systematically understand the regulatory mechanisms operating in a cancer cell. For this task, it is important to discover a set of co-expressed genes with functions, representing a so-called functional gene module, because co-expressed genes tend to be co-regulated by the same regulators, including transcription factors, microRNAs, and copy number aberrations. Several algorithms have been used to identify such gene modules, including hierarchical clustering and non-negative matrix factorization. Although these algorithms have been applied to many microarray datasets, only a few systematic analyses of these algorithms have been performed for RNA-sequencing (RNA-Seq) data to date. Although gene expression levels determined based on microarray and RNA-Seq datasets tend to be highly correlated, the expression levels of some genes differ depending on the platforms used for analysis, which may result in the construction of different gene modules for the same samples. Here, we compare several module detection algorithms applied to both microarray and RNA-seq datasets. We further propose a new functional gene module detection algorithm (FGMD), which is based on a hierarchical clustering algorithm that was modified to reflect actual biological observations, including the fact that a single gene may be involved in multiple biological pathways. Application of existing algorithms and the new FGMD algorithm to breast cancer and ovarian cancer datasets from The Cancer Genome Atlas showed that the FGMD algorithm had the best performance for most of the functional pathway enrichment tests and in the transcription factor enrichment test. We expect that the FGMD algorithm will contribute to improving the identification of functional gene modules related to cancer.

## Introduction

Cancer is primarily a genetic disease, and aberrations in the DNA sequence and expression of multiple genes in cancer cells have been detected. Although many studies have identified the candidate genes that play important roles in cancer development, the combinatorial effects of a set of genes, which may constitute a functional gene module, have not yet been fully elucidated. Indeed, co-expressed genes across multiple samples have been frequently observed to interact with each other and to participate in the same biological functions or signaling pathways [1]. A co-expressed gene network is generally represented as an undirected graph, where the nodes represent genes and the edges represent the strength of relationships between them. Functional gene modules often correspond to dense subnetworks of the co-expressed gene network; thus, it is important to identify genes that are highly correlated in the network, because cancer genes generally regulate other genes, directly or indirectly. A co-expressed gene network is generally represented as an undirected graph, where the nodes represent genes and the edges represent the strength of relationships between them. Several co-expression networks have been constructed to date and used to identify biomarkers such as hub genes and disease candidate genes [2]. For example, GeneFriends [3] was developed to predict novel candidate disease genes by expanding known disease genes based on a co-expression network. Moreover, co-expressed genes are likely to be co-regulated by the same regulators [4] such as a transcription factor (TF) or microRNA (miRNA). Genes under the control of the same regulator or that are located in the same copy number aberration regions tend to exhibit similar expression patterns [5–8].

An important step toward identifying such gene regulatory modules is to incorporate information on the relationships among genes. For example, some genes are directly regulated by TFs while others are indirectly regulated by TFs by interacting with the directly regulated genes. Thus, when analyzing gene expression data, it is important to detect functional gene modules in which the genes are highly inter-correlated and play a key role in cancer-related functions and pathways.

Because gene expression levels can now be readily measured using microarray and RNA-sequencing (RNA-Seq), in the present study, we aimed to systematically compare functional gene modules identified from these two platforms using widely adopted algorithms. Furthermore, we developed a new algorithm to detect functional modules, and compared its performance to that of the existing algorithms.

We first systematically compared gene expression data obtained by microarray and RNA-Seq from breast invasive carcinoma (BRCA) samples available in The Cancer Genome Atlas (TCGA) project [9]. Zhao et al. [10] reported that although the two platforms showed similar tendencies in revealing expression changes for the same samples, some genes showed significant differential expression between the microarray and RNA-Seq datasets. Therefore, we computed several statistics representing the characteristics of the two datasets, including coefficient of variation, total connectivity, density, centralization, and heterogeneity [11], and measured the correlations of gene expression levels between the data generated from microarray and RNA-Seq.

Second, we developed a functional gene module detection (FGMD) algorithm that starts with seed gene pairs, expands these pairs to construct modules, combines similar modules, and finally splits them into specialized modules by utilizing hierarchical clustering [12]. Although hierarchical clustering has been widely adopted for detecting functional gene modules, in these previous models, one gene is assumed to only belong to one module in a mutually exclusive manner. Therefore, this method does not reflect the biological reality that a single gene is often involved in several biological functions or signaling pathways. Nevertheless, hierarchical clustering has several advantages. First, it easily establishes a variety of distance metrics that can be used to quantitatively determine the relationships among genes. Second, the number of clusters can be determined using algorithms such as the “Dynamic Tree Cut” algorithm [13], which can identify nested clusters and is robust to outliers. Thus, to derive the FGMD algorithm, we built a hierarchical clustering model that was extended to incorporate the fact that one gene can be involved in many modules.

Third, we systematically compared the performance of several algorithms for detecting functional gene modules based on module evaluation criteria for microarray and RNA-Seq platforms. The SAMBA biclustering algorithm [14] finds subgraphs exhibiting consistent patterns in a subset of conditions. Here, densely connected subgraphs of the bipartite graph are considered as modules. Although this approach provides many significant modules enriched in several pathways, the performance is highly dependent on the normalization method adopted, data type, and size of the dataset. The non-negative matrix factorization (NMF) algorithm [15] factorizes a gene expression matrix into the basis matrix and the coefficients matrix. The membership of genes for a given module is determined through the basis matrix. Even though NMF is widely used for module detection, it is difficult to find the optimal rank *r* that determines the number of modules, and the performance is highly dependent on the rank *r*. Weighted correlation network analysis (WGCNA) [16] constructs co-expression networks and the co-expression similarity raised to a power. Then, hierarchical clustering with the “Dynamic Tree Cut” algorithm is used to construct gene modules of the highly correlated genes. Even though this approach can identify several biologically significant modules, the genes in the module tend to show a relative lack of functional relevance. Moreover, in contrast to the SAMBA biclustering algorithm and NMF algorithm, WGCNA does not reflect the fact that one gene can be involved in multiple modules.

Finally, we constructed ovarian cancer (OVC) modules using gene and isoform expression data. We first compared the gene and isoform expression data of RNA-Seq for an OVC dataset from TCGA [17]. We then demonstrate that some validated gene–gene interaction (GGI) pairs can only be captured based on isoform expression. In addition, we applied the FGMD algorithm to the gene and isoform expression data and compared the respective modules constructed with the two datasets.

## Materials and methods

### Datasets and preprocessing

**Fig 1:**
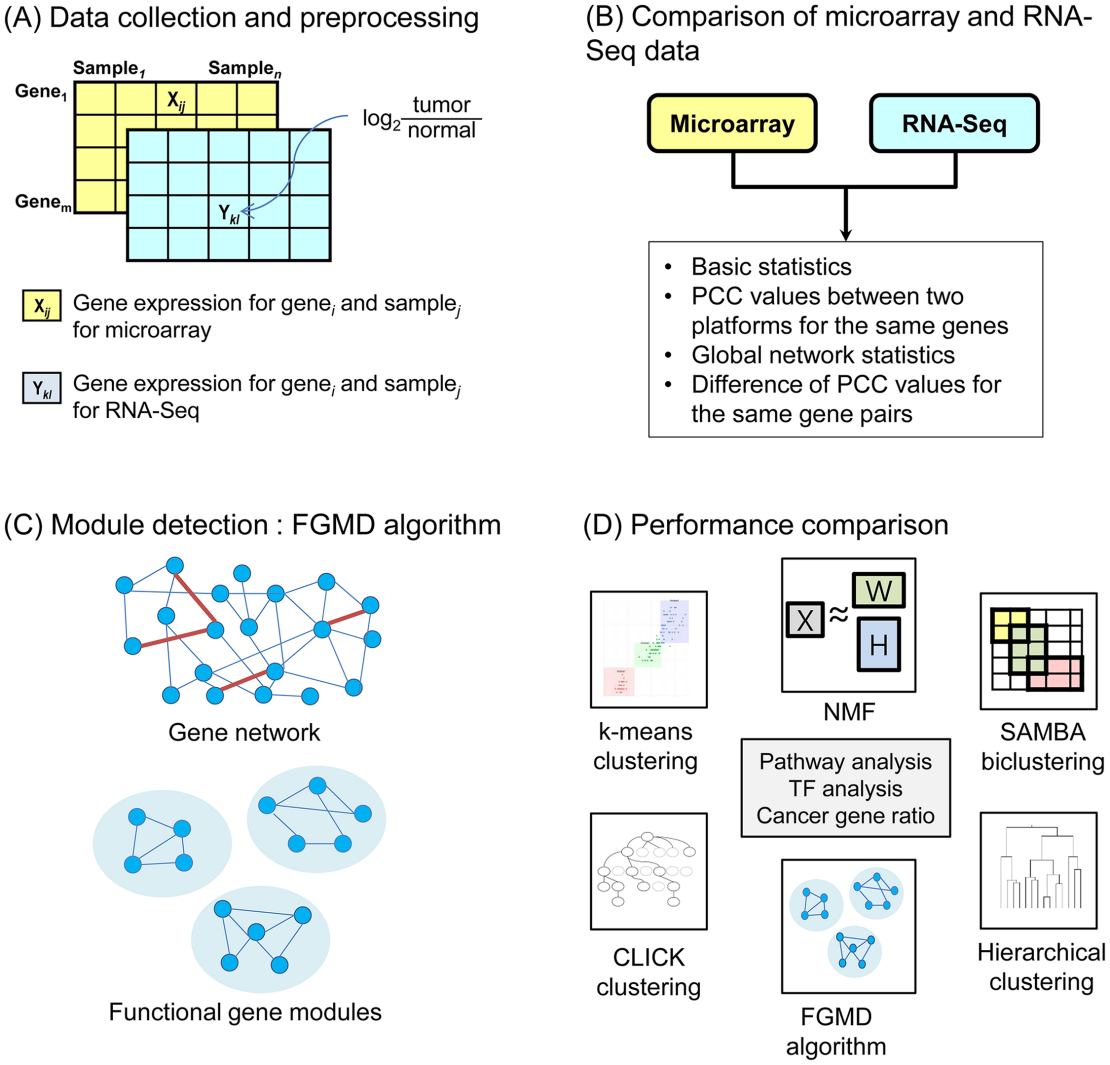
Overview of our approach. (A) Collect gene expression data obtained from microarray and RNA-Seq platforms for paired samples, and calculate the log2 ratios between tumor samples and the average of normal samples. (B) Compare gene expression data of the microarray and RNA-Seq datasets. (C) Construct FGMD modules using the microarray and RNA-Seq gene expression data. Further details are provided in Fig 2. (D) Compare the modules constructed by FGMD to those constructed by other methods. PCC, Pearson correlation coefficient; TF, transcription factor.

For the breast cancer data, we collected gene expression data for 492 tumor samples and 53 unmatched normal samples obtained from both microarray and RNA-Seq platforms published in TCGA. Microarray and RNA-Seq data were generated from an Agilent G4502A and Illumina HiSeq_RNA_Seq platform, respectively, and we used level-3 data in TCGA. To understand the relationship between the two platforms, we extracted 14,352 genes that were common to the microarray and RNA-Seq datasets. Because the RNA-Seq dataset contained many genes with zero RPKM values [18], we replaced the zero values with the minimum non-zero value. Then, gene expression values were normalized to the log2 ratio between values in tumor samples and the average values of unmatched normal samples to obtain the relative abundance values (Fig 1(A)).

For the ovarian cancer data, we collected RNA-Seq data for 291 tumor samples from TCGA generated by the Illumina HiSeq_RNA_SeqV2 platform, which provides isoform-level expression as well as gene-level expression. Thus, we used 20,531 genes and 73,599 isoforms. Because there were no available expression data for normal tissue samples, tumor expression values were not normalized to those of normal samples in this case.

### Comparison of gene expression data from microarray and RNA-Seq

We calculated the basic statistics of the gene expression values from the microarray and RNA-Seq datasets, such as the minimum, maximum, average, and range. Similar to Iancu et al. [11], we computed the absolute values of the Pearson correlation coefficient (PCC) matrix between all gene pairs and further examined gene pairs with a large difference in absolute PCC values between the microarray and RNA-Seq data. Let *a*_*ij*_ = |*corr*(*x*_*i*_, *x*_*j*_)|, which indicates the connection strength. For each gene, the total node connectivity is computed as *k*_*i*_ = ∑_*j*_ *a*_*ij*_, representing the sum of the absolute PCC values between gene *i* and all other genes. The coefficient of variation was calculated as the standard deviation of the connection strengths divided by the average of the connection strengths. In addition, global network statistics were computed using the following equations:

$$\begin{array}{c} Density=\frac{\sum_{i}\sum_{j}a_{ij}}{n(n-1)}\\ Centralization=\frac{max(k)}{n}-Density\\ Heterogeneity=\frac{\sqrt{variance(k))}}{mean(k))} \end{array}$$

where *k* = {*k*_1_, *k*_2_, *k*_3_, …, *k*_*n*_}, and *n* represents the number of genes.

### Comparison of gene and isoform expression data of RNA-Seq

We examined the minimum, 25th percentile, median, 75th percentile, and maximum expression values, and filtered out genes and isoforms whose expression values were close to zero. In addition, we computed the PCC values of isoform expression levels between gene pairs in GGIs from the Human Protein Reference Database (HPRD) [19]. Then, we compared the PCC values of gene expression for the same gene pairs in the GGI. Note that for isoform expression, we used the maximum value of the PCC values among all combinations of isoform pairs corresponding to the genes.

### FGMD algorithm

**Fig 2:**
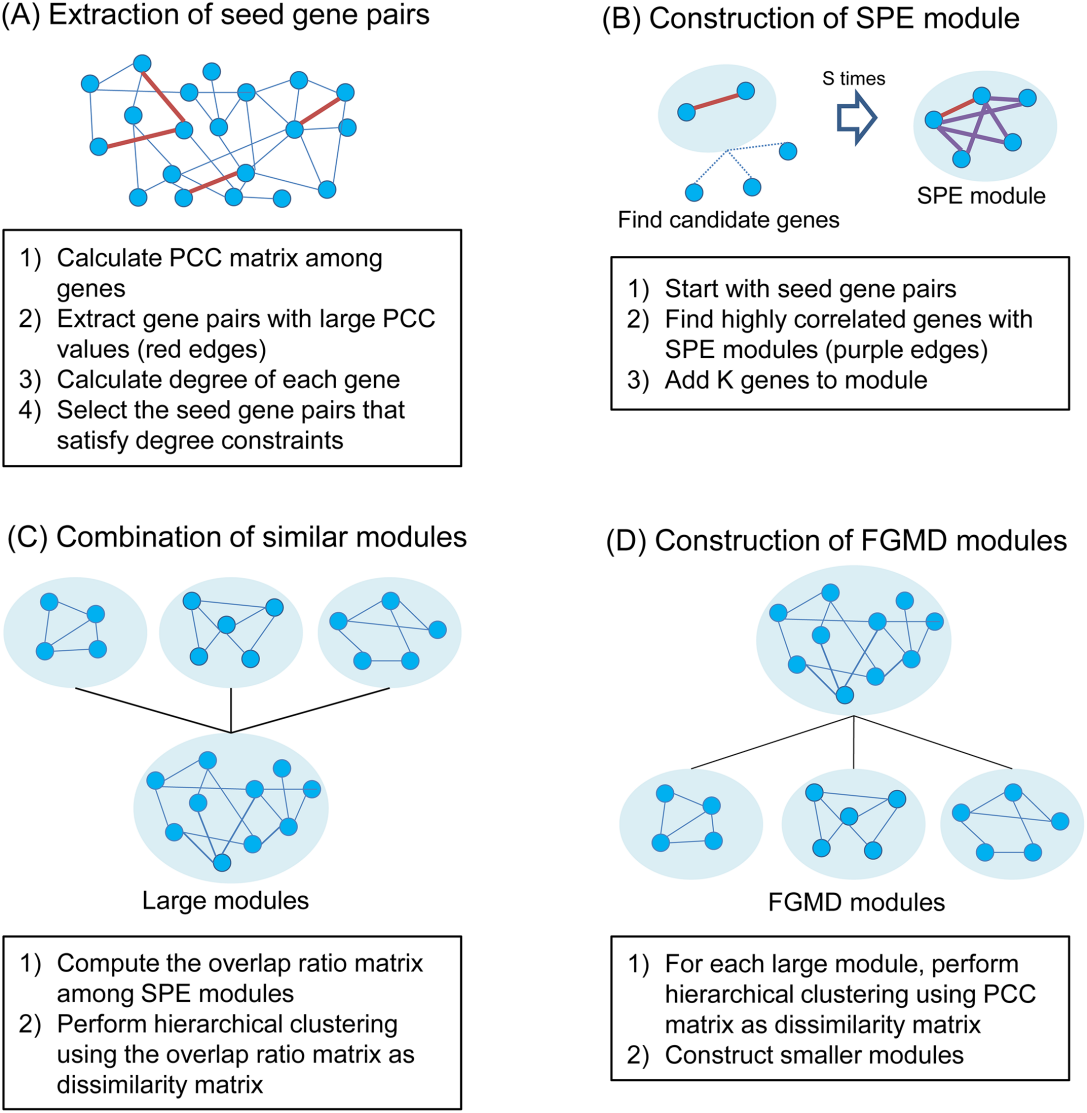
Overview of the FGMD algorithm. (A) Extract seed gene pairs that show high Pearson correlation coefficient (PCC) values and satisfy degree constraints. (B) Construct seed gene pair expansion (SPE) modules by expanding seed gene pairs. (C) Combine similar modules based on the overlap ratio using hierarchical clustering with “Dynamic Tree Cut.” (D) Construct FGMD modules by applying hierarchical clustering to each large module.

We here present the FGMD algorithm, which extends the hierarchical clustering algorithm to incorporate the actual biological observation that a gene is often involved in multiple functions or pathways (Fig 1(C)). We first selected seed gene pairs and constructed modules by expanding the gene pairs using a greedy approach. We then performed a merging process and a split process to identify functional gene modules. Fig 2 provides an overview of the FGMD algorithm and details are described below.

*Step 1. Extraction of seed gene pairs based on PCC values.* A PCC matrix is constructed, where the similarity between genes is measured using an absolute PCC value. We select gene pairs with the top *S*% of PCC values as seed gene pairs (*S* = 0.005), and construct a seed gene pair network, where the nodes are genes and the edges are connected between seed gene pairs; one gene can belong to multiple pairs. In the seed gene pair network, we compute the degree of a gene as the number of connected genes through the seed gene pair edges. If one gene is connected to too many genes or to only a few genes, the gene (along with its connected edges) will be filtered out because genes with high connectivity to other genes generate similar modules, while genes related to few genes result in small-sized modules. Specifically, genes in the seed gene pair network are filtered out if the degree of the genes is smaller than *D*_*min*_ = 5 or larger than *D*_*max*_ = the degree of the top 0.1% of genes when genes are ordered by the degrees in the network. Note that even if one gene in a pair is filtered out, the paired gene can remain if it still satisfies the degree condition above.

*Step 2. Seed gene pair expansion.* We next construct modules based on seed gene pairs. A seed gene pair is regarded as an initial module. Then, *k* candidate genes, representing the number of genes added to the module at a time (here, *k* = 10), are selected that satisfy the following conditions:

(i) The similarity between genes in a module and a candidate gene is determined to be the minimum PCC value (*P*_*min*_) among all PCC values between the candidate gene and genes in the modules.
(ii) *P*_*min*_ is higher than the module threshold *M*. The module threshold *M* is determined as the top 1% of PCC values in the PCC matrix.
(iii) Genes not satisfying degree constraints are excluded to reduce redundancy among modules.
(iv) Among genes satisfying (i), (ii), and (iii), *k* genes with the largest *P*_*min*_ values are selected as candidate genes.

The above procedure is iterated until there are no more genes satisfying the conditions. The final constructed modules are termed seed gene pair expansion (SPE) modules. In the expansion process, one gene can be selected by multiple seed gene pairs so that several modules may overlap with each other.

*Step 3. Merging similar modules.* In some cases, the overlap between some SPE modules might be too large. We can therefore measure the overlap ratio between modules using the following equation and construct an overlap ratio matrix.

$$\begin{array}{c} overlap\_ratio\left(m_{1},m_{2}\right)=\frac{|m_{1}\cap m_{2}|}{|m_{1}\cup m_{2}|}, \end{array}$$

where *m*_1_ and *m*_2_ are the numbers of genes in the two modules. We used a hierarchical clustering method to control the overlap ratio. The dissimilarity matrix is (1—overlap ratio matrix), and highly overlapped modules are grouped into the same cluster using hierarchical clustering followed by Dynamic Tree Cut [13], which starts with a few large clusters in the dendrogram, and adaptively iterates for cluster decomposition and combination. Then, we construct new modules by merging genes in the same cluster. These modules are referred to as “large modules.”

*Step 4. Construction of functional gene modules.* Finally, we reconstruct the modules by dividing the large modules into smaller modules. For each large module, genes in the module are clustered using the hierarchical clustering algorithm with (1 − PCC of expression levels between two genes) as the dissimilarity values. Then, highly correlated genes are grouped into a cluster using the Dynamic Tree Cut algorithm. As a result, “FGMD modules” are constructed, where genes overlap among modules with a proper overlap ratio. Source codes for the FGMD algorithm are available at http://gcancer.org/FGMD.

### Module validation

For validation, we employed several module evaluation criteria, including in-module PCC values, overlap ratio among modules, enrichment of functional pathways, enrichment of the targets of TFs, and the ratio of cancer-related genes.

We performed enrichment tests of the modules for Gene Ontology (GO) biological processes, Kyoto Encyclopedia Gene and Genome (KEGG) pathways, and BioCarta pathways to validate the functional relevance of the identified modules. First, we downloaded information of these pathways from the Gene Set Enrichment Analysis (GSEA) database (http://www.broadinstitute.org/gsea). Then, we excluded biological functions and signaling pathways associated with less than three genes and more than 300 genes to exclude any modules that were either too specific or too general. Second, for each term, we computed a *p*-value using a hypergeometric test and adjusted it to a *q*-value based on the Benjamini-Hochberg multiple comparison correction method; terms with a *q*-value < 0.05 were considered statistically significant. The TF enrichment test was performed according to the same method employed for the functional pathway enrichment test. Allocco et al. [4] observed that co-expressing genes tended to be regulated by the same TFs. Hence, if some modules are enriched with target genes regulated by TFs, these modules are likely to be regulated by the same TF in either a direct or indirect manner.

We collected information on BRCA-related genes from the Breast Cancer Database (http://www.breastcancerdatabase.org), which contains BRCA genes altered at the DNA, RNA, and protein levels as well as drug-induced altered genes. The dataset originally contained information on 2,958 BRCA genes. Among them, we extracted 2,544 BRCA genes in common with our gene set, indicating that 17.7% of the genes in our dataset are BRCA-related genes. We then computed the ratio of BRCA genes in the modules and computed the *p*-values. 567 cancer gene census (CGC) genes and 2,027 cancer-related genes were collected from Cancer Gene Census (http://cancer.sanger.ac.uk/census) [20] and the Bushman lab (http://www.bushmanlab.org/links/genelists) [20–23], respectively.

In addition, we evaluated the statistical significance of the genes in the modules based on the null hypothesis that the average PCC values of genes in modules are similar to the average PCC values of genes in random modules of the same size. For each module, we performed the following test.

(i) Calculate module_*avg*_, the average of the PCC values of genes in modules without diagonal elements.
(ii) Construct random gene modules by randomly selecting genes from a gene expression matrix.
(iii) Calculate random_*avg*(*i*)_, the average of the PCC values of genes in the *i*_*th*_ random module without diagonal elements.
(iv) Repeat (ii) and (iii) *N* times.
(v) Calculate the *p*-value using the following equation.
  $$\begin{array}{c} \text{p-value}=\frac{\sum_{i=1}^{N}I\left(module_{avg}<random_{avg}\left(i\right)\right)}{N} \end{array}$$

We used *N* = 1,000, and the *p*-values were corrected to *q*-values to address multiple comparison issues using the Bonferroni correction.

### Performance comparisons

The performance of five existing module detection algorithms was compared to that of the FGMD algorithm using the same gene expression data. Because some algorithms, including FGMD, generate too small or too large modules, we removed modules containing less than 10 genes or more than 300 genes. Similarly, for the isoform modules, only modules containing more than 10 and less than 500 isoforms were used. Modules generated by the algorithms are referred to as *M*1–*M*9. Modules *M*1–*M*8 are described in the following sections, and *M*9 refers to modules generated by the FGMD algorithm.

#### Hierarchical clustering: *M*1 and *M*2

Hierarchical clustering [12] iteratively combines small clusters into larger clusters. Because the Euclidean distance and PCC are widely used metrics for constructing a dissimilarity matrix, we constructed the modules using these two metrics as the dissimilarity matrix, referred to as *M*1 and *M*2, respectively. We performed hierarchical clustering using the ‘hclust’ package in R.

#### K-means clustering: *M*3

In the K-means algorithm [24], the parameter *k*, representing the number of clusters, needs to be pre-determined. Here, we set *k* to 1000, which could generate many singleton clusters. Based on the module selection criteria, the singleton clusters were deleted and the remaining clusters were used as modules (*M*3). We performed K-means clustering using EXPANDER software [25].

#### CLICK clustering: *M*4

CLICK clustering [26] utilizes graph-theoretic and statistical techniques, where the data are modeled as a weighted graph. In this graph, the vertices are genes and the edge weights represent the degree of similarity between genes. The graphs are partitioned into components based on the minimum cut algorithm, and similar clusters were iteratively merged (*M*4). We performed CLICK clustering using EXPANDER software [25].

#### SAMBA biclustering: *M*5

The SAMBA biclustering algorithm [14] finds subgraphs exhibiting consistent patterns in a subset of conditions. One of the key advantages of the biclustering algorithm is to allow overlaps among modules, reflecting the biological fact that one gene may be related to several functions. This algorithm models gene expression data as a bipartite graph, where one part is a set of genes and the other is a set of samples. Edges are linked between genes and conditions if the expression values of a subset of genes are significantly altered in a subset of the samples, representing high correlations. As a result, subgraphs of the bipartite graph are output as functional gene modules (*M*5). We performed the SAMBA biclustering using EXPANDER software [25].

#### NMF: *M*6 and *M*7

NMF [15] factorizes a matrix *V* into two matrices, *W* and *H*, where all three matrices have only non-negative values. NMF attempts to find a small number of metagenes that represent a linear combination of *m* genes [15]. A gene expression matrix with size *m* × *n* is factorized into matrix *W* with size *m* × *r* and into matrix *H* with size *r* × *n*, where *r* is the rank. In these matrices, each column *r* in *W* defines metagenes and column *n* in *H* represents the gene expression of metagenes for corresponding samples. Because the BRCA gene expression dataset includes negative values, we converted these to non-negative values according to the procedure outlined in Kim and Tidor [27]. We created two matrices, containing only positive or negative values from the original gene expression matrix, respectively. Other values in the two matrices were set to zero, and the negative matrix was converted to the positive matrix by multiplying by -1. Finally, the two matrices were combined to *m* by a 2*n* matrix that only contains positive values. We performed NMF using the “NMF” package in R. This package implements the NMF algorithm based on Kullback-Leibler divergence, which was previously used to reduce the dimensions of gene expression data [28]. In determining the membership of genes for each module, we used a method proposed by Kim and Park [29]. First, the basis matrix *W* was converted to probability scores to easily interpret the contribution of each gene to each module. Then, we selected genes whose values were higher than $\hat{u}\text{(median)}+3\hat{\sigma }\text{(median absolute deviation)}$ and whose maximum contribution in the row of the basis matrix *W* was greater than the median of all values.

In the NMF algorithm, it is important to appropriately choose the rank *r*, which defines the size of the matrix and thus determines the number of modules. However, the estimation process to identify the optimal rank *r* that can reconstruct a matrix to most closely match the original matrix is time-consuming and could generate many singleton modules. Hence, we performed NMF using ten *r* values (*r* = 10, 20, 30, 40, 50, 60, 70, 80, 90, and 100), and selected one rank *r* based on the sum of the ratio of modules enriched in GO, KEGG, BioCarta, and TF, and the ratio of cancer genes (*M*6). We define this rank *r* as the best rank.

In addition, we combined all modules generated by a set of the ten rank values using a greedy approach (*M*7). First, we computed the overlap ratio between modules. Second, we found two modules that represent the maximum overlap and merged them until no modules with an overlapping ratio > 0.5 remained.

#### WGCNA: *M*8

WGCNA [16] can be used to construct modules of highly correlated genes. First, a gene network is constructed based on the absolute values of correlation coefficients. Then, hierarchical clustering with the “Dynamic Tree Cut” algorithm is used for module detection. To handle large datasets, all nodes are divided into large clusters called “blocks”, and then hierarchical clustering is applied to each block and the resulting modules are merged, whose eigengenes are highly correlated. As a result, WGCNA outputs modules of highly correlated genes (*M*8). We performed the WGCNA algorithm using the “WGCNA” package in R.

## Results

### Comparison of BRCA gene expression data from microarray and RNA-Seq

We compared the expression profiles of the 14,352 genes identified from the microarray and RNA-Seq datasets. The result showed a broader range in the expression values from RNA-Seq than those from the microarray (S1 Fig). In addition, we computed the absolute PCC values among all gene pairs from both datasets. When comparing the absolute PCC values for gene pairs using a pairwise *t*-test, RNA-Seq showed higher PCC values than microarray with a *p*-value < 2.2e-16 (S2(A) Fig). In addition, we computed the difference in absolute PCC values between the microarray and RNA-Seq data for the same gene pairs. The average of all differences was 0.06, implying that most of the relationships between genes were similarly represented in the two platforms (S2(B) Fig). However, some gene pairs showed a significant difference (e.g., 43,339 pairs had a difference in PCC values > 0.5), implying that the relationship between some genes might only be detectable in one platform. The average PCC value for the same genes common to both microarray and RNA-Seq was 0.683, demonstrating the similar tendency of identifying expression changes in the two platforms (S3 Fig). Moreover, our results showed that a gene network based on expression values measured using RNA-Seq has higher density and variability, although the gene network from the microarray showed slightly higher centralization than that from RNA-Seq. Details are described in the Supplementary Information (S1 File).

### Comparison of OVC gene and isoform expression from RNA-Seq

We next compared the gene and isoform expression profiles for the OVC dataset. We first checked the range of expression values for 20,531 genes and 73,599 isoforms in 291 samples. Considering the data distribution, we filtered 25% and 50% of the lowly expressed genes and isoforms, respectively, based on average expression values that were close to zero, and additionally filtered genes or isoforms whose values were zero for more than half of the samples. After filtering, we used 15,374 genes and 35,535 isoforms for the construction of FGMD modules. We also compared the distribution of PCC values for genes in validated GGI pairs using gene expression and isoform expression data (S4(A) Fig). The result showed that the PCC values of the isoform expression levels were slightly higher than those of gene expression levels. Of the 31,006 GGI pairs, 1,046 showed large differences in PCC values (> 0.3) (S4(B) Fig). This result demonstrates that although many studies regularly combine expression data for a set of isoforms of a single gene, using the isoform expression itself can help to construct different functional gene modules. Further details are described in the Supplementary Information (S1 File).

### Construction of BRCA FGMD modules

#### Extraction of seed pairs based on absolute PCC values

For the microarray, we set the seed threshold and the module threshold as the default values: top 0.005% (0.828) and top 1% (0.402) of the absolute PCC values, respectively. For each gene, we computed the degree of genes, representing the number of linked genes, via the seed pair edges. For the two genes in seed pairs, we restricted the degree of genes from 5 to 70 (0.1% of the degree of all genes). As a result, 3,469 seed pairs consisting of 320 genes were extracted (Step 1). For RNA-Seq, we set the seed threshold and the module threshold as the top 0.005% (0.842) and top 1% (0.456) of the absolute PCC values, respectively. We then computed the degree of genes for each gene. For the two genes in the seed pairs, we restricted the degree of genes from 5 to 73. As a result, 3,335 seed pairs consisting of 350 genes were extracted (Step 1).

#### Seed gene pairs expansion and construction of FGMD modules

As described in the Methods section, we expanded seed gene pairs using the greedy approach and constructed SPE modules. Then, we constructed large modules by combining similar modules based on the overlap ratio among modules. For applying the Dynamic Tree Cut algorithm, we used default parameters (maxTreeHeight = 1, deepSplit = TRUE, and minModuleSize = 50).

For the microarray data, the algorithm began with 3,469 seed gene pairs as the initial SPE modules. Then, the SPE modules were expanded by determining the 10 genes with the highest PCC values, which were added to the SPE modules. This process was repeated until no further genes could be added. As a result, 3,469 SPE modules were constructed (185 genes per module, Step 2). Second, we computed the overlap ratio matrix between the 3,469 SPE modules. Hierarchical clustering was then performed, and nine large modules were constructed (361 genes per module, Step 3). Similarly, for the RNA-Seq data, 3,335 seed gene pairs were first considered as the initial SPE modules, which were expanded to construct 3,335 SPE modules (209 genes per modules, Step 2). The combination of similar SPE modules resulted in the construction of 16 large modules (289 genes per module, Step 3).

We divided the large modules into functional gene modules. For the microarray data, we performed hierarchical clustering with the absolute PCC matrix for each of the nine large modules. As a result, 32 FGMD modules were constructed (Step 4). For the RNA-Seq data, hierarchical clustering with the PCC matrix for each of the 16 large modules resulted in the construction of 44 FGMD modules (Step 4).

### Comparison of the performance of different algorithms for the BRCA modules

We compared the modules generated by the existing methods (*M*1–*M*8) and the FGMD algorithm (*M*9) using the BRCA data. The genes in modules identified with microarray and RNA-seq are listed in S1 and S2 Tables, respectively. In addition, the highest rank *r* in NMF was selected based on the results shown in S3 Table. As shown in Table 1, most of the modules exhibited statistical significance in a permutation test. In particular, the NMF (*M*6 and *M*7) and FGMD (*M*9) modules showed relatively high in-module PCC values for both the microarray and RNA-Seq data, demonstrating that the expression levels of genes in these modules are highly correlated. The number of genes can differ depending on the algorithm adopted, since most algorithms generate only highly correlated clusters.

**Table 1:** Module statistics of BRCA modules using microarray and RNA-Seq data derived with different algorithms. “Mean of # of genes in modules” represents the average of the number of genes in the modules. “# of unique genes” represents the number of genes for all modules without duplication. “Average occurrence of genes” indicates the number of modules in which a given gene is included. “Mean of in-module PCC values” represents the average of PCC values among genes in the modules. “# of significant modules” represents the number of modules whose PCC value among genes is higher than that of random modules based on the permutation test.

BRCA modules using microarray									
Statistics	*M*1	*M*2	*M*3	*M*4	*M*5	*M*6	*M*7	*M*8	*M*9
# of modules	311	305	86	90	206	10	40	37	32
Mean of # of genes in modules	43.52	42.59	63.69	79.94	112.41	100.4	73	57.05	88.01
# of unique genes	13,535	12,989	5,477	7,195	3,813	1,004	1,351	2,111	963
Ratio of module duplication	0	0	0	0	0.036	0	0.022	0	0.072
Average occurrence of genes	1	1	1	1	6.07	1	2.16	1	2.92
Mean of in-module PCC values	0.233	0.300	0.401	0.270	0.291	0.531	0.526	0.460	0.660
# of significant modules	219	269	85	90	193	10	40	37	32
BRCA modules using RNA-Seq									
Statistics	*M*1	*M*2	*M*3	*M*4	*M*5	*M*6	*M*7	*M*8	*M*9
# of modules	301	323	48	56	291	7	21	84	44
Mean of # of genes in modules	47.84	40.70	89.17	91.41	89.60	44.14	51.86	54.08	90
# of unique genes	13,196	13,147	4,280	5,119	4,094	309	580	4,543	1,172
Ratio of module duplication	0	0	0	0	0.025	0	0.030	0	0.070
Average occurrence of genes	1	1	1	1	6.37	1	1.88	1	3.37
Mean of in-module PCC values	0.292	0.353	0.363	0.317	0.237	0.643	0.707	0.467	0.694
# of significant modules	226	276	45	56	215	6	20	84	44

We performed functional pathway enrichment tests using GO biological processes, and the KEGG and BioCarta pathways to validate the functional relevance of the constructed modules. Although the numbers of unique genes included in the modules differed depending on the algorithm adopted, the *q*-value of each module in the enrichment test was calculated by the hypergeometric test, where the background number of genes was set to the number of human genes included in the datasets (14,352 and 20,531 genes for breast cancer and ovarian cancer, respectively). We considered pathways with a *q*-value < 0.05 as statistically significant. The results are shown in Table 2 for the KEGG pathways and S4 Table for GO biological processes and the BioCarta pathways. The enriched pathways are listed in S5–S13 Tables for the microarray data and in S14–S22 Tables for the RNA-Seq data. Module evaluation criteria included the ratio of enriched modules and the number of enriched terms per module. Compared to the existing algorithms, our FGMD algorithm showed the highest performance for all functional pathway enrichment tests in the microarray data, and for all enrichment tests except for the ratio of enriched modules in KEGG pathways for the BRCA RNA-Seq data.

**Table 2:** KEGG pathway and TF enrichment tests for BRCA modules using microarray and RNA-Seq data derived with different algorithms. “# of enriched modules” indicates the number of modules enriched with at least one KEGG pathway. “Ratio of enriched modules in KEGG” is computed by “# of enriched modules in KEGG” divided by “# of modules.” “# of enriched KEGG terms per module” is computed by “# of enriched KEGG terms” divided by “# of modules.” “# of enriched modules” indicates the number of modules enriched with at least one TF. “Ratio of enriched modules in TF” is computed by “# of enriched modules in TF” divided by “# of modules.” “# of enriched TF per module” is computed by “# of enriched TF” divided by “# of modules”.

BRCA modules using microarray									
Statistics	*M*1	*M*2	*M*3	*M*4	*M*5	*M*6	*M*7	*M*8	*M*9
# of modules	311	305	86	90	206	10	40	37	32
# of enriched modules in KEGG	51	62	27	18	97	52	20	11	29
Ratio of enriched modules in KEGG	0.164	0.203	0.314	0.2	0.471	0.8	0.5	0.297	0.906
# of enriched KEGG terms	136	163	110	43	340	52	192	37	270
# of enriched KEGG terms per module	0.437	0.534	1.797	1.989	2.782	5.2	4.8	1.0	8.438
# of enriched modules in TFs	34	53	24	28	63	6	13	13	24
Ratio of enriched modules in TFs	0.109	0.174	0.279	0.311	0.306	0.600	0.325	0.351	0.75
# of enriched TFs	178	387	154	179	573	22	80	133	116
# of enriched TFs per module	0.572	1.269	1.797	1.989	2.782	2.200	2.000	3.594	3.625
Ratio of BRCA genes	0.182	0.180	0.280	0.147	0.371	0.224	0.185	0.231	0.295
Ratio of CGC genes	0.035	0.037	0.043	0.037	0.044	0.042	0.046	0.041	0.057
Ratio of Cancer genes	0.125	0.122	0.168	0.109	0.172	0.151	0.126	0.123	0.189
BRCA modules using RNA-Seq									
Statistics	*M*1	*M*2	*M*3	*M*4	*M*5	*M*6	*M*7	*M*8	*M*9
# of modules	301	323	48	56	291	7	21	84	44
# of enriched modules in KEGG	56	66	20	11	117	6	11	13	36
Ratio of enriched modules in KEGG	0.186	0.204	0.417	0.196	0.402	0.857	0.523	0.154	0.818
# of enriched KEGG terms	166	189	86	32	567	39	98	43	443
# of enriched KEGG terms per module	0.551	0.585	1.792	0.571	0.513	5.571	4.666	0.512	10.068
# of enriched modules in TFs	45	63	19	19	86	3	10	22	37
Ratio of enriched modules in TFs	0.15	0.206	0.396	0.339	0.296	0.429	0.476	0.261	0.84
# of enriched TFs	202	452	293	105	758	13	67	117	291
# of enriched TFs per module	0.67	1.474	6.104	1.875	2.605	1.857	3.19	1.392	6.614
Ratio of CGC genes	0.038	0.035	0.037	0.033	0.034	0.032	0.035	0.039	0.055
Ratio of BRCA genes	0.185	0.180	0.234	0.159	0.257	0.304	0.314	0.159	0.281
Ratio of Cancer genes	0.126	0.121	0.156	0.111	0.14	0.168	0.169	0.104	0.193

We also performed a TF enrichment test to determine the modules co-regulated by TFs. Table 2 shows the results of the TF enrichment tests for each method. Enriched TFs are listed in S23 Table for the microarray data and in S24 Table for the RNA-Seq data. The module evaluation criteria for this test included the ratio of enriched modules and the number of enriched TFs per module. Again, the FGMD modules showed the highest performance in the TF enrichment test compared to the other methods.

In addition, we compared the ratio of BRCA genes identified with each algorithm (Table 2). The details of BRCA genes in each module are provided in S25 and S26 Tables. For the microarray data, the FGMD modules provided a higher ratio of BRCA genes than the other methods, except for the SAMBA modules (*M*5). However, this high ratio of BRCA genes using SAMBA was due to a large number of overlaps among modules. Considering only the unique genes of the FGMD modules and SAMBA modules to determine the ratio of BRCA genes for the microarray data, the FGMD modules contained a higher ratio of BRCA genes (288 of 963, 29.9%) compared to those in the SAMBA module (1,032 of 3,813, 27.0%). Furthermore, for the RNA-Seq data, the FGMD modules showed the highest ratio of BRCA genes compared to the other methods, except for the NMF modules (*M*6 and *M*7). However, the numbers of NMF modules were too small, consisting of only 309 genes for *M*6 and 580 genes for *M*7, whereas the FGMD modules consisted of 1,172 genes. Therefore, although the ratio of BRCA genes was slightly lower with the FGMD method, these results demonstrate the high potential of FGMD to find novel BRCA genes or functional pathways.

**Fig 3:**
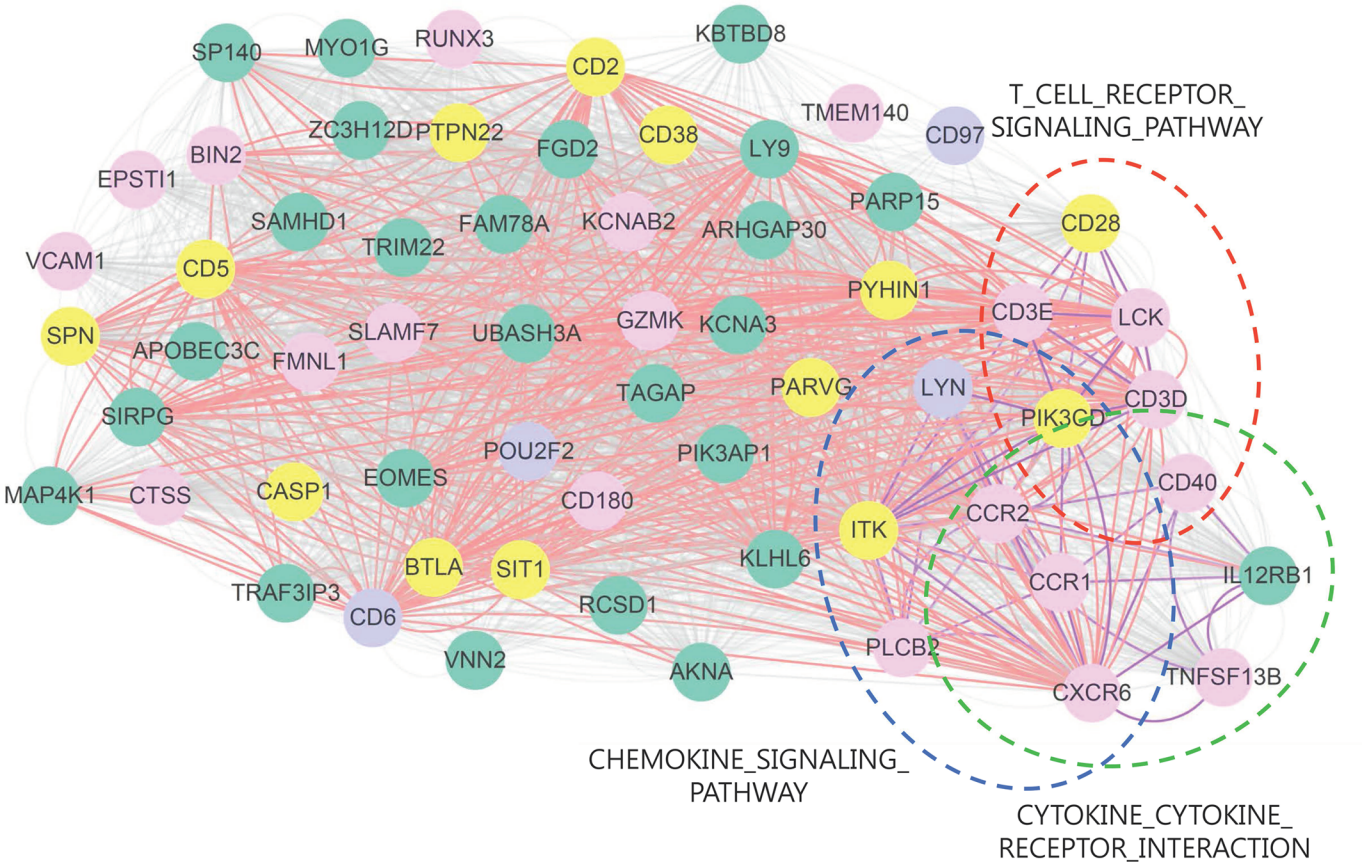
Network presentation of BRCA module 40 using RNA-Seq. In this network, nodes represent genes: pink nodes indicate BRCA genes supported by the Breast Cancer Database (http://www.breastcancerdatabase.org), yellow nodes indicate BRCA genes supported by DigSee [29], and purple nodes indicate cancer genes obtained from http://www.bushmanlab.org/links/genelists. A red line indicates that the PCC value between genes is larger than 0.842 (the seed threshold) and a gray line indicates that the PCC value between genes is larger than 0.650 (0.1%). A purple line indicates that the linked genes are enriched together with at least one function, and a dotted ellipse represents genes included in the enriched pathway. For example, *CD28, CD3E, LCK, CO3D, PIK3CD*, and *CD40* are enriched with at least one function together (T-cell receptor signaling pathway).

Fig 3 illustrates a BRCA module (module 40) that contains 62 genes, representing a highly correlated dense network. In this module, 20 genes (shown in pink) are likely to be BRCA-related genes in the Breast Cancer Database (http://www.breastcancerdatabase.org). Thirteen genes (in yellow) are listed as potentially BRCA-related genes in DigSee [29], with supporting statements from the literature. In addition, three of the seven enriched KEGG pathways, T cell receptor signaling pathway, chemokine signaling pathway, and cytokine–cytokine receptor interaction, were represented in this module. These pathways have been previously related to BRCA [30–32], and most of the genes involved in these pathways have been shown to be BRCA-related genes. Thus, we expect that *LYN* and *IL12RB1* are likely to be related to BRCA, because they are highly correlated with BRCA-related genes and belong to the same pathways. Indeed, there is support for an association of these two genes with BRCA in the literature [33, 34]. These observations support that the genes in modules interact with each other and play critical roles in BRCA at the pathway level.

Our results demonstrated some key disadvantages of the NMF approach for detecting functional gene modules. First, the performance is highly dependent on the rank *r*, and determining the optimal rank *r* or a set of rank *R* is time-consuming and optimal performance is not guaranteed. Second, the NMF modules (*M*6) contained a small number of modules, with only one gene belonging to one module. In addition, because we selected the best rank *r* in *R* based on the sum of the ratios of enriched modules in the GO processes, KEGG and BioCarta pathways, TF, and the ratio of BRCA genes, high performance is necessarily achieved. Although the NMF modules (*M*6) have the main advantage of incorporating more information compared to the other approaches, the performance of this method was not the best.

### Construction of FGMD modules for OVC

#### Extraction of seed pairs based on absolute PCC values

For the OVC gene expression data, we set the seed threshold and the module threshold as the top 0.005% (0.832) and top 1% (0.365) of the absolute PCC values, respectively. For each gene, we computed the degree of genes and restricted the degree of genes from 5 to 68 for the two genes in seed pairs. As a result, 3,507 seed pairs consisting of 469 genes were extracted (Step 1).

For the OVC isoform expression data, we set the seed threshold and the module threshold as the top 0.005% (0.756) and top 1% (0.347) of absolute PCC values, respectively. For each gene, we computed the degree of isoforms. For the two isoforms in seed pairs, we restricted the degree of isoforms from 5 to 130. As a result, 18,729 seed pairs consisting of 1,759 isoforms were extracted (Step 1).

#### Seed pairs expansion and construction of FGMD modules

For the gene expression data, 3,507 seed gene pairs were used as the initial SPE modules, and expansion of the SPE modules resulted in the construction of 3,507 SPE modules (151 genes per module, Step 2). Then, based on hierarchical clustering, 15 large modules (217 genes per module, Step 3) were constructed. Similarly, for the isoform expression data, after starting with 18,729 seed pairs as the initial SPE modules, 18,729 SPE modules (205 isoforms per module, Step 2) and 88 large modules (306 isoforms per module, Step 3) were constructed.

Hierarchical clustering for each of the 15 and 88 large modules resulted in the construction of 28 and 245 FGMD modules for the gene expression and isoform expression data, respectively (Step 4).

### Comparison of the performance of different algorithms for OVC modules

We compared the modules generated by the existing methods (*M*1–*M*8) and the FGMD algorithm (*M*9) using the OVC data. As shown in Table 3, most of the modules exhibited significance in the permutation test. The specific genes and isoforms in the modules for each method are listed in S27 and S28 Tables, respectively. Note that the best rank *r* in NMF was selected based on the results shown in S29 Table.

**Table 3:** Module statistics of OVC modules using gene and isoform expression data with different algorithms. “Mean of # of genes (or isoforms) in modules” represents the average of the number of genes (or isoforms) in the modules. “# of unique genes (or isoforms)” represents the number of genes (or isoforms) for all modules without duplication. “Average occurrence of genes (or isoforms)” indicates the number of modules in which a given gene (or isoform) is included. “Mean of in-module PCC values” represents the average of PCC values among genes (or isoforms) in the modules. “# of significant modules” represents the number of modules whose PCC value among genes (or isoforms) is higher than that of random modules based on the permutation test.

OVC modules using gene expression									
Statistics	M1	M2	M3	M4	M5	M6	M7	M8	M9
# of modules	327	336	24	115	94	32	166	105	28
Mean of # of genes in modules	43.98	41.41	63.83	91.41	180.94	29.44	32.48	50.10	91.57
# of unique genes	14,381	13,914	1,532	10,512	1,817	942	1,553	5,260	989
Ratio of module duplication	0	0	0	0	0.094	0	0.012	0	0.060
Average of occurrence of genes	1	1	1	1	9.36	1	3.471	1.0	2.592
Mean of in-module PCC values	0.176	0.343	0.296	0.251	0.094	0.368	0.261	0.459	0.653
# of significant modules	230	330	18	115	18	32	140	105	28
OVC modules using isoform expression									
Statistics	M1	M2	M3	M4	M5	M6	M7	M8	M9
# of modules	754	800	27	149	17	23	127	133	245
Mean of # of isoforms in modules	44.78	40.95	86.44	166.02	32.05	29.39	35.13	74.00	91.29
# of unique isoforms	33,763	32,756	2,334	24,737	451	676	1,722	9,842	4,822
Ratio of module duplication	0.170	0	0	0	0.017	0	0.013	0	0.022
Average of occurrence of isoforms	1	1	1	1	1.21	1	2.591	1	4.638
Mean of in-module PCC values	0.170	0.324	0.294	0.228	0.234	0.450	0.281	0.445	0.598
# of significant modules	485	781	26	149	13	23	112	133	245

We performed the functional pathway enrichment test to validate the functional relevance of the modules. Table 3 and S30 Table show the comparison results of the functional pathway enrichment tests of gene and isoform expression data for each method. Enriched pathways are listed in S31–S39 Tables for gene expression and in S40–S48 Tables for isoform expression. The results showed that our FGMD algorithm had the highest performance for all functional pathway enrichment tests for both the gene and isoform expression data.

**Fig 4:**
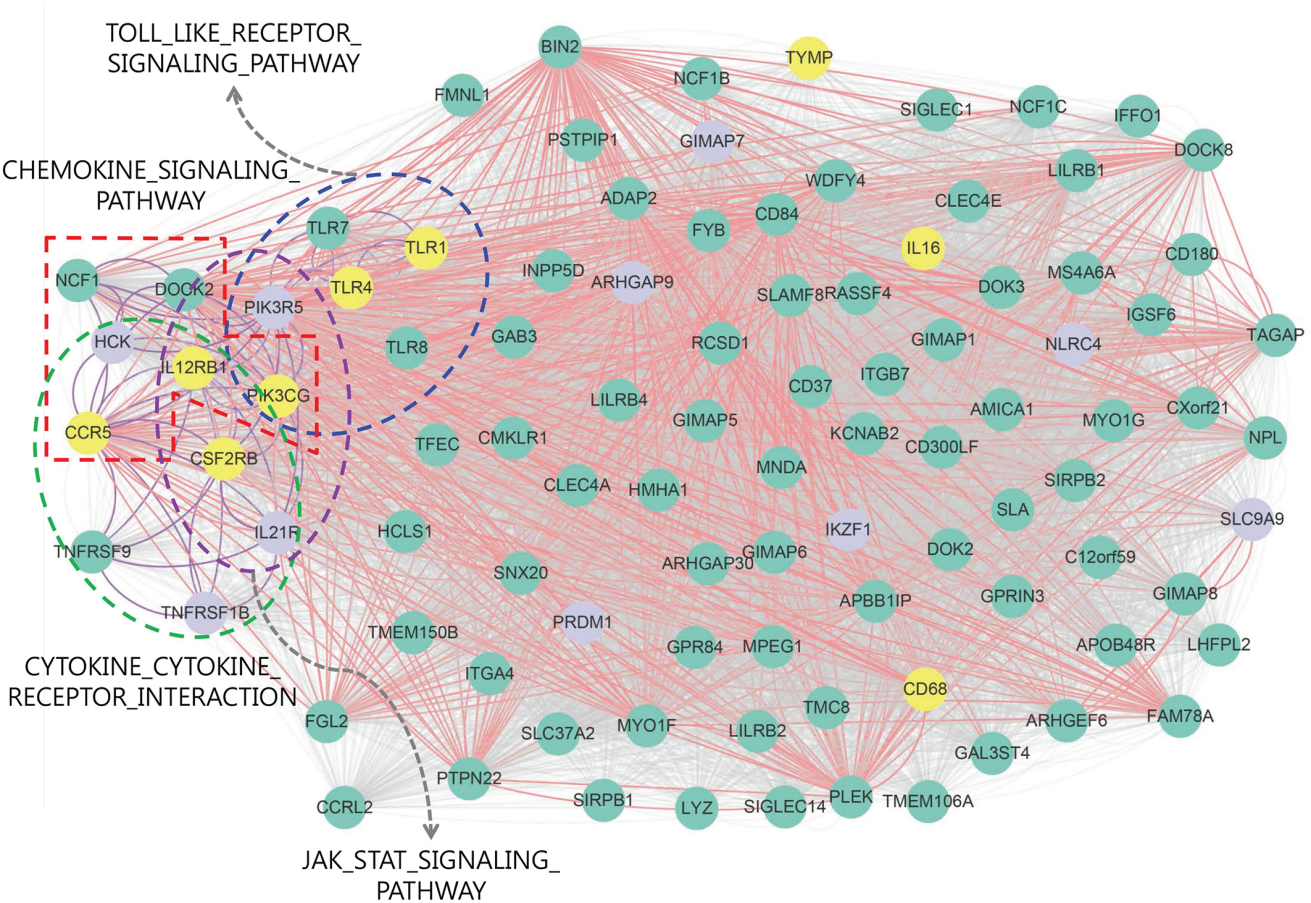
Network presentation of OVC module 3 using gene expression data. In this network, nodes represent genes: yellow nodes indicate OVC genes supported by DigSee [29], purple nodes indicate cancer genes obtained from http://www.bushmanlab.org/links/genelists, and green nodes indicate the remaining genes. A red line indicates that the PCC value between genes is larger than 0.832 (the seed threshold) and a gray line indicates that the PCC value between genes is larger than 0.579 (0.1%). A purple line indicates that the linked genes are enriched together with at least one function, and a dotted ellipse includes genes enriched in the same pathway.

Fig 4 illustrates OVC module 3 that contains 96 genes, representing a highly correlated dense network. In this module, nine genes (in yellow) were identified as OVC-related genes in DigSee [29], with supporting evidence from the literature. In addition, four of the five enriched KEGG pathways, Toll-like receptor signaling pathway, chemokine signaling pathway, JAK-STAT signaling pathway, and cytokine–cytokine receptor interaction, were represented in the module. These pathways have been reported to be related to OVC [35–38]. Moreover, most of the OVC genes were represented intensively in these pathways. In particular, the genes enriched in these pathways show high potential to be OVC-related genes. Several studies have also supported that *TNFRSF9, TNFRSF1B, PIK3R5, HCK, TLR7*, and *TLR8* were related to OVC [35, 39–42]. Furthermore, genes in this module are highly correlated with each other, and thus might play important roles in OVC.

We also performed the TF enrichment test to check modules that contain genes co-regulated by TFs. Table 4 also shows the results of the TF enrichment test for each method. Enriched TFs are listed in S49 Table for gene expression data and in S50 Table for isoform expression data. Based on the module evaluation criteria of the ratio of enriched modules and the number of enriched TF per modules, the FGMD modules showed the highest performance in the TF enrichment test. On the other hand, the ratios of CGC genes and cancer genes were not higher compared to those of some other methods. Thus, the overall rankings of all comparing methods are evaluated in the Discussion section.

**Table 4:** Comparison of KEGG pathway and TF enrichment tests for OVC modules using gene and isoform expression data. “# of enriched modules” indicates the number of modules enriched with at least one KEGG pathway. “Ratio of enriched modules in KEGG” is computed by “# of enriched modules in KEGG” divided by “# of modules.” “# of enriched KEGG terms per module” is computed by “# of enriched KEGG terms” divided by “# of modules.” “# of enriched modules” indicates the number of modules enriched with at least one TF. “Ratio of enriched modules in TF” is computed by “# of enriched modules in TF” divided by “# of modules.” “# of enriched TF per module” is computed by “# of enriched TF” divided by “# of modules”.

OVC modules using gene expression									
Statistics	M1	M2	M3	M4	M5	M6	M7	M8	M9
# of modules	327	336	24	115	94	32	166	105	28
# of enriched modules in KEGG	32	46	12	10	38	11	26	14	19
Ratio of enriched modules in KEGG	0.098	0.136	0.500	0.087	0.404	0.344	0.157	0.133	0.679
# of enriched KEGG terms	119	113	50	14	87	39	190	58	126
# of enriched KEGG terms per module	0.363	0.336	2.083	0.583	0.926	1.219	1.144	0.552	4.5
# of enriched modules in TF	36	46	7	17	35	8	36	28	19
Ratio of enriched modules in TF	0.110	0.136	0.291	0.147	0.372	0.25	0.157	0.266	0.679
# of enriched TF terms	126	113	97	150	313	77	408	295	81
# of enriched TF terms per module	0.385	0.336	4.04	1.304	3.329	2.438	2.458	2.810	2.892
Ratio of CGC genes	0.033	0.032	0.048	0.031	0.027	0.035	0.033	0.037	0.034
Ratio of cancer genes	0.122	0.117	0.154	0.106	0.139	0.157	0.158	0.101	0.128
OVC modules using isoform expression									
Statistics	M1	M2	M3	M4	M5	M6	M7	M8	M9
# of modules	754	800	27	149	17	23	127	133	245
# of enriched modules in KEGG	72	72	13	29	3	6	25	23	118
Ratio of enriched modules in KEGG	0.095	0.09	0.481	0.194	0.176	0.261	0.197	0.173	0.481
# of enriched KEGG terms	222	153	63	76	9	19	113	91	510
# of enriched KEGG terms per module	0.294	0.192	2.333	0.510	0.529	0.826	0.99	0.684	2.081
# of enriched modules in TF	117	72	11	55	0	9	29	55	118
Ratio of enriched modules in TF	0.155	0.09	0.407	0.369	0	0.391	0.197	0.414	0.481
# of enriched TF	521	153	250	761	0	103	604	761	510
# of enriched TF per module	0.691	0.192	9.259	5.107	0	4.478	4.7559	5.721	2.081
Ratio of CGC genes	0.038	0.041	0.054	0.038	0.074	0.062	0.041	0.04	0.037
Ratio of cancer genes	0.124	0.129	0.167	0.122	0.17	0.17	0.162	0.125	0.155

### Comparison of BRCA FGMD modules using microarray and RNA-Seq data

**Fig 5:**
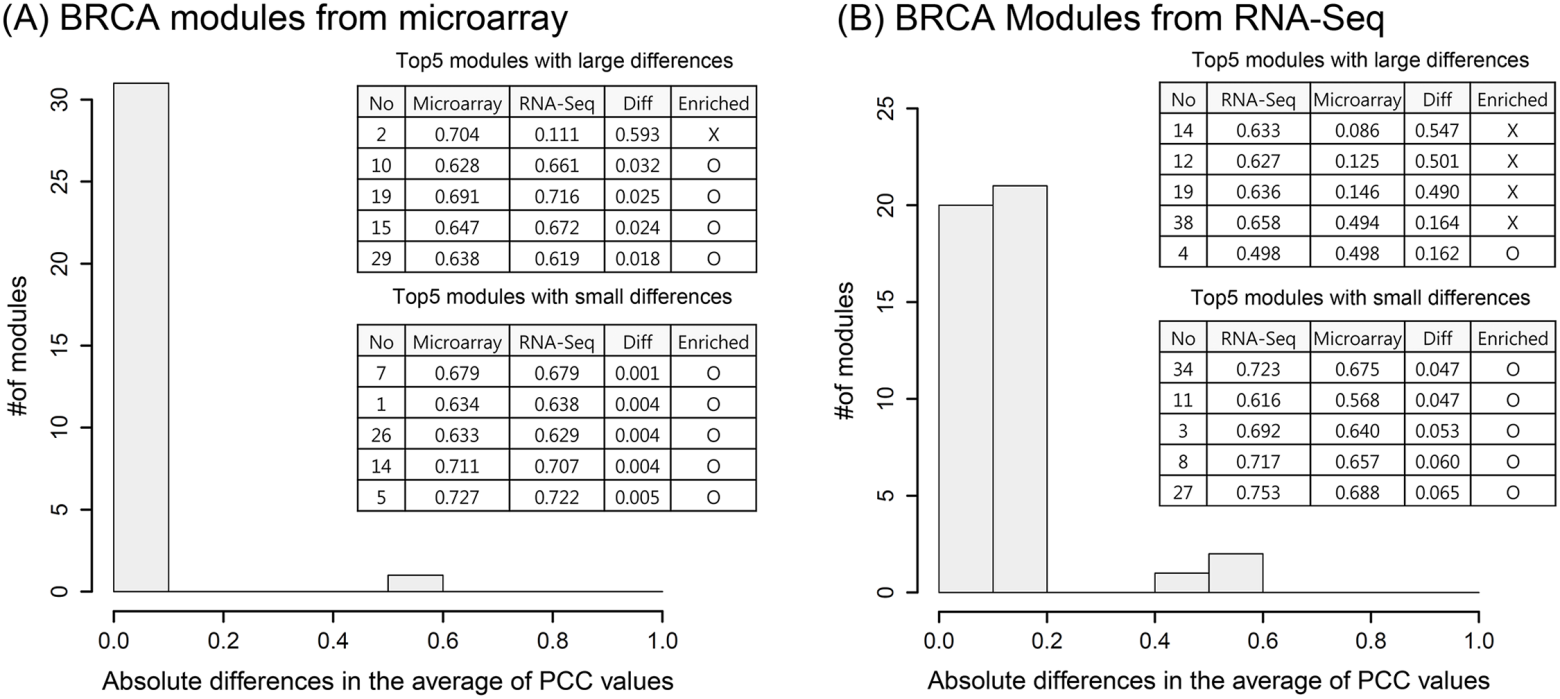
Differences in the average of PCC values for BRCA genes in modules identified by microarray and RNA-Seq. Distribution of the absolute differences in the average of PCC values between genes in microarray (A) and RNA-Seq (B) modules. In the table, “No” represents the identifier of a module constructed using microarray (A) or RNA-Seq (B), “Microarray” represents the average of PCC values for genes in the module using expression data from the microarray, “RNA-Seq” represents the average of PCC values for genes in the module using expression data from RNA-Seq, and “Diff” represents the difference between the previous two values. “Enriched” indicates whether the module is enriched with GO terms, or KEGG or BioCarta pathways.

For BRCA, we compared modules based on the microarray and RNA-Seq data constructed by the FGMD algorithm. Fig 5 shows the distribution of the number of modules according to the absolute difference in the average of PCC values of the genes in the two platforms. For gene pairs in a module identified by microarray, the PCC values of expression levels were calculated, and then the PCC values of all gene pairs were averaged. For the same gene pairs, the average PCC of expression levels obtained from the RNA-seq data was calculated. The difference between the two average PCC values is shown in Fig 5(A). Similarly, using gene pairs in a module identified by RNA-seq, the average PCC value was obtained. For the same gene pairs, the average of the PCC values of expression levels obtained from the microarray was calculated. The differences between the two average PCC values are shown in Fig 5(B). For most modules, the same gene pairs showed high PCC values in the other platform (microarray or RNA-Seq); however, some modules contained genes that were highly correlated only in one platform. Among the modules obtained from the microarray, only module 2 showed large differences with RNA-Seq and was not enriched with GO biological processes, or with KEGG and BioCarta pathways. Among modules from RNA-Seq with large differences from those of the microarray, modules 14, 12, 15, and 38 were not enriched in any pathway. The fact that 36 of 44 modules were enriched suggests that modules that are captured only in one platform might not be functionally associated. This finding implies that the two platforms can be utilized as a validation tool of modules constructed by one or the other platform.

### Comparison of OVC FGMD modules using gene and isoform expression data

For OVC, we compared the modules generated from the gene and isoform expression levels constructed by the FGMD algorithm. For most modules, those constructed according to gene expression data could also be constructed using the isoform expression data, because gene expression levels tend to be highly correlated with at least one of the isoforms of the gene. In addition, isoforms of the same gene were frequently correlated, and were therefore included in the same module. Furthermore, when computing the overlap ratio between modules constructed by gene and isoform expression data, genes in the modules constructed with the gene expression data highly overlapped with at least one of the modules constructed by the isoform expression data.

**Fig 6:**
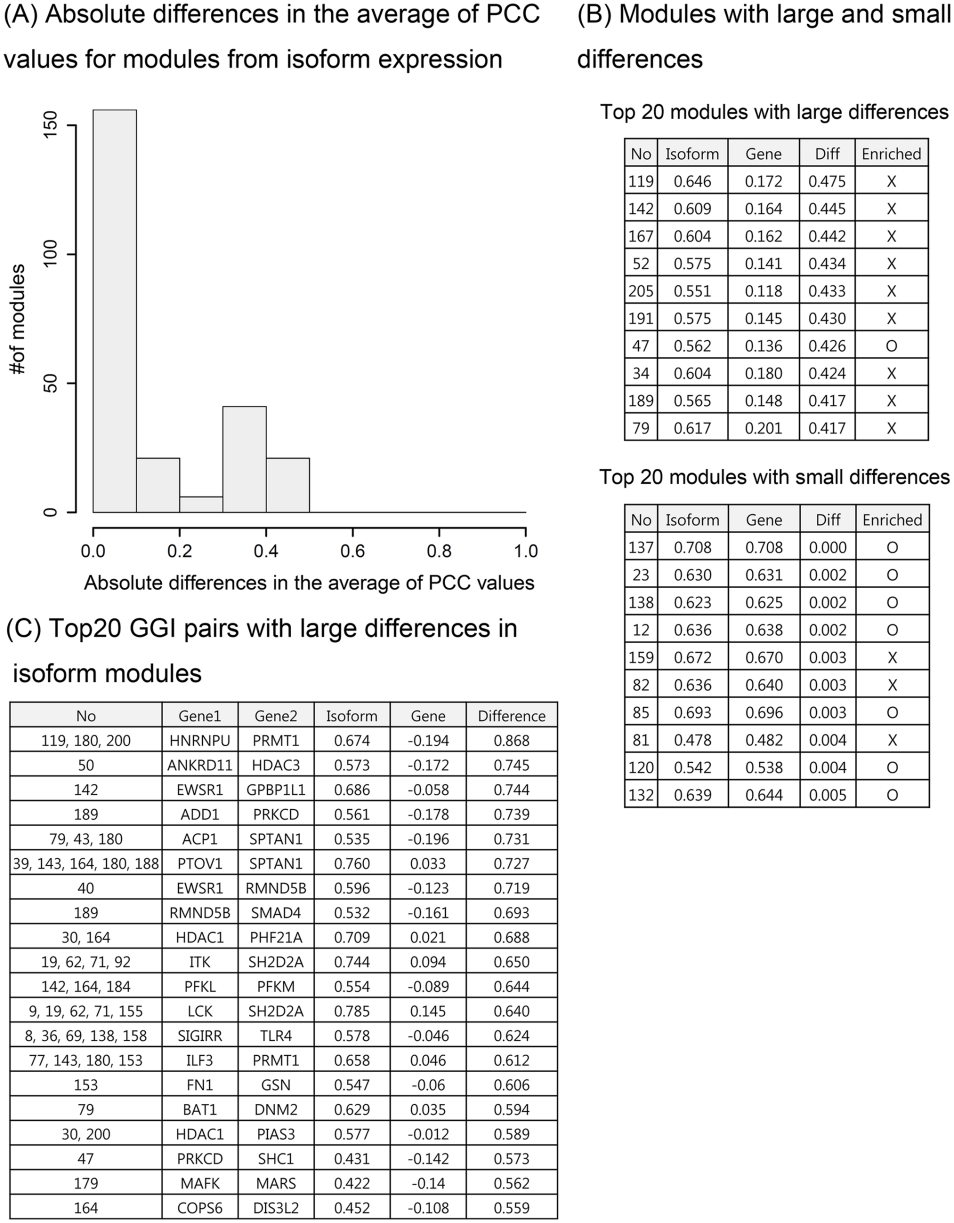
Differences in the average of PCC values for genes in modules identified using gene and isoform expression levels for OVC. Distribution of the absolute differences in the average of PCC values between gene and isoform expression data for isoform expression modules (A). Modules with large and small differences are shown (B). Among the validated GGI pairs included in the isoform modules, some GGI pairs were not highly correlated at the gene level. The GGI pairs with large differences at the isoform level and at the gene level are shown in (C). In the table, “No” represents the identifier of a module, “Gene” represents the average of PCC values using gene expression, “Isoform” represents the average of PCC values using isoform expression, and “Difference” represents the difference between the two previous values.

However, the isoform expression levels differed from the gene expression levels in several modules. Fig 6(A) shows the distribution of the absolute differences in the average PCC values between gene-level and isoform-level expression for genes in the isoform expression modules. Fig 6(B) shows isoform modules with large and small differences compared to the gene-level expression. Among the 10 modules with large differences, only one module was enriched in functional pathways, whereas among the 10 modules with small differences, seven modules were enriched in functional pathways. This finding shows that although isoform expression data can be used to construct modules that cannot be constructed with gene expression data, most of these modules did not have functional relevance. However, some functionally related modules could only be constructed based on isoform expression data, such as module 47 that was enriched in the cellular component assembly and positive regulation of translation categories. Furthermore, we compared the PCC values from the gene and isoform expression data for GGI pairs in the isoform modules. For most pairs, these were highly correlated, but some gene pairs were captured only in the isoform expression data. Fig 6(C) shows the GGI pairs that had large absolute differences in PCC values between the gene and isoform data.

## Discussion

In this study, we developed a new functional gene module detection algorithm, FGMD, to facilitate investigations of various regulatory mechanisms in cancer, and applied it to the BRCA gene expression data from the microarray and RNA-Seq datasets and OVC gene and isoform expression data from RNA-Seq datasets. Comparison of the BRCA gene expression data from the microarray and RNA-Seq datasets showed that most genes had a similar tendency of altered expression in breast cancer tissues in the two platforms, with high PCC values. This implies that most of the relationships between genes can be similarly captured in the two platforms. Comparison of the FGMD modules constructed using microarray and RNA-Seq data showed 963 and 1,172 unique genes, respectively, and 674 genes were common between platforms, implying that the modules generated from the two platforms consisted of similar genes, which are consequently enriched in the same pathways. However, some of the modules from the microarray and RNA-Seq data also consisted of different genes. Thus, adoption of both platforms could facilitate the discovery of novel cancer genes, functions, and pathways that would otherwise only be detected in one platform.

Comparison of OVC gene and isoform expression data from RNA-Seq showed that the same gene tended to show similar alterations in both datasets. Thus, most of the validated GGI pairs could be captured, regardless of whether gene or isoform expression levels were considered. However, some of the GGI pairs were only captured by the isoform expression data. In addition, compared with the FGMD modules constructed by gene and isoform expression data, it was more difficult to construct some modules identified using isoform expression data using gene expression data. This indicates that isoform expression analysis can be helpful to best understand GGIs, because different isoforms of the same gene might interact with different genes. Thus, isoform expression plays an important role in the discovery of novel cancer genes, functions, and pathways, and for understanding the detailed molecular mechanisms of cancer.

We compared the performance of the FGMD algorithm (*M*9) with that of other existing algorithms (*M*1–*M*8). Because we used several evaluation criteria, we summarized the performance of these methods by ranking these methods for each criterion (the ratios of enriched modules in GO terms, KEGG pathways, BioCarta pathways, and TFs, and the ratios of cancer genes, CGC genes, and BRCA genes). All ranking information is described in S51 Table. The FGMD method had the highest average rank for the BRCA microarray, BRCA RNA-seq, and OVC gene expression datasets, and had the second highest average rank for the OVC isoform expression data. Note that the K-means clustering method (*M*3) had the highest average rank for OVC isoform expression data and was ranked equivalently to the FGMD method for the OVC gene expression data. However, the K-means clustering method ranked 5^th^ and 4^th^ for the BRCA microarray and BRCA RNA-seq datasets, respectively. Considering all four datasets, the performance of the FGMD method was the best on average. Hierarchical clustering (*M*1 and *M*2), CLICK clustering (*M*4), and WGCNA (*M*8) showed the lowest performances overall. In addition, the SAMBA biclustering algorithm (*M*5) showed relatively higher performance. The SAMBA algorithm outputs many modules enriched with several pathways, and consequently shows good potential for finding novel cancer genes and functional pathways. However, depending on the method of normalization of the raw data, the generated modules may have different performances. Thus, appropriate normalization methods should be selected depending on the type and size of the dataset. Moreover, the sizes of the identified modules are highly dependent on the size of the dataset. When the size of the dataset increases, the size of the modules increases, generating large-sized modules. Indeed, when the SAMBA algorithm was applied to the isoform expression data from the OVC RNA-Seq dataset, many modules containing more than 1500 isoforms were generated. Although these modules might contain many functional pathways, they often lack modularity, requiring adjustment of the size of the data to construct reliable functional gene modules. The NMF algorithm (*M*6) showed slightly higher performance than the SAMBA biclustering algorithm. However, it is challenging and time-consuming to determine the optimal rank *r* using this method.

One disadvantage of the FGMD algorithm is that it takes a long time to expand the seed pairs, especially with a high number of seed pairs. We measured the running time of each method using the BRCA gene expression data from microarray and a comparison is shown in S52 Table. The FGMD algorithm took longer than hierarchical clustering (*M*1 and *M*2), CLICK clustering (*M*4), SAMBA biclustering (*M*5), and WGCNA (*M*8), but was faster than K-means clustering (M3) and NMF (*M*6 and *M*7). Hence, more efficient seed selection and expansion methods are required in future work to optimize this method.

When a large module was divided into small modules using the the Dynamic Tree Cut algorithm, we used the default parameter values. Because a ‘minModuleSize’ parameter determining the size and number of small modules affects the performance of the FGMD algorithm, we compared the performance using five parameter values: 20, 30, 50 (default), 70, and 100. When the parameter value increased, the number of modules was reduced and the performance increased. However, when the ‘minModuleSize’ value was greater than or equal to 50 (the default value), there was no difference in the ratio of enriched modules in GO terms and KEGG pathways. Thus, we used the default parameters when we applied the FGMD algorithm to the BRCA and OVC datasets. Details are described in S53 Table.

In the FGMD algorithm, although small modules belonging to different larger modules can overlap, the small modules in the same large module do not overlap. This is because small modules are generated using the hierarchical algorithm. To explore the possibilities of further performance improvement, we developed another approach that allows for overlap among the small modules generated from the same large module. First, for each small module *m*_*i*_ in a large module *M*_*j*_, we attempt to select genes *g*_*k*_ that are in the large module but not in the small module *m*_*i*_ (*g*_*k*_ ∈ *M*_*j*_ and *g*_*k*_ ∉ *m*_*i*_) and are highly correlated with genes in *m*_*i*_. We calculate the average PCC value of gene expression levels between genes in *m*_*i*_ and each gene *g*_*k*_ (*g*_*k*_ ∈ *M*_*j*_ and *g*_*k*_ ∉ *m*_*i*_). Then, we add a gene with the highest PCC value to the small module *m*_*i*_, and continue to add genes with the next highest PCC values until adding more genes no longer improves the average PCC value among genes in the small module *m*_*i*_. We applied this new approach to the OVC and BRCA datasets, and the comparison results with and without overlaps of small modules are shown in S54 Table. Although the overlapping ratios increased more than doubled, the performances were improved by allowing for overlaps among small modules. In particular, the improvement in the OVC isoform expression dataset was notable because it outperformed all other methods, including the K-means clustering algorithm (*M*3) that showed the best performance in the OVC isoform expression dataset. The source codes of this modified approach are also available at http://gcancer.org/FGMD, providing the choice between the overlap ratio of modules and more functionally related modules. Note that even though gene duplication between small modules is not allowed in the FGMD algorithm, gene duplication was nevertheless reflected through the overlapping of large modules. Highly related genes can be used to construct the final modules when they belong to the same large modules. At the same time, other large modules can increase the opportunity to construct final modules that overlap with other final modules.

## Conclusion

The FGMD algorithm represents a new framework for utilizing existing algorithms. Although the FGMD algorithm is based on hierarchical clustering to combine similar modules and split them, other clustering approaches can be used as an alternative with some modification at each step. In addition, our algorithm relies only on gene expression data to detect functional gene modules without incorporating information on gene regulation or GGIs. Thus, application of our proposed algorithm to other types of biological data such as miRNA expression, copy number aberrations, TF-binding sites, gene–miRNA binding information, and GGIs might enable the construction of gene-regulatory modules reflecting various biological processes. In conclusion, the FGMD algorithm will contribute toward improving the detection of functional gene modules, which can help to gain new insight into the underlying mechanisms of cancer.

## Supporting information

S1 Table Genes in BRCA modules for microarray data. (XLSX)

S2 Table Genes in BRCA modules for RNA-Seq data. (XLSX)

S3 Table Comparison of the performance of BRCA modules constructed by NMF for determining the rank r. (XLSX)

S4 Table Comparison of enrichment tests for GO terms and BioCarta pathways for BRCA modules. (XLSX)

S5 Table GO, KEGG, and BioCarta enrichment tests for BRCA modules constructed by hierarchical clustering with the Euclidean distance metric (M1) using microarray data. (XLSX)

S6 Table GO, KEGG, and BioCarta enrichment tests for BRCA modules constructed by hierarchical clustering with the PCC distance metric (M2) using microarray data. (XLSX)

S7 Table GO, KEGG, and BioCarta enrichment tests for BRCA modules constructed by K-means clustering with the PCC distance metric (M3) using microarray data. (XLSX)

S8 Table GO, KEGG, and BioCarta enrichment tests for BRCA modules constructed by CLICK clustering (M4) using microarray data. (XLSX)

S9 Table GO, KEGG, and BioCarta enrichment tests for BRCA modules constructed by SAMBA biclustering (M5) using microarray data. (XLSX)

S10 Table GO, KEGG, and BioCarta enrichment tests for BRCA modules constructed by NMF (M6) using microarray data. (XLSX)

S11 Table GO, KEGG, and BioCarta enrichment tests for integrated BRCA modules constructed by NMF (M7) using microarray data. (XLSX)

S12 Table GO, KEGG, and BioCarta enrichment tests for integrated BRCA modules constructed by WGCNA (M8) using microarray data. (XLSX)

S13 Table GO, KEGG, and BioCarta enrichment tests for BRCA modules constructed by FGMD (M9) using microarray data. (XLSX)

S14 Table GO, KEGG, and BioCarta enrichment tests for BRCA modules constructed by hierarchical clustering with the Euclidean distance metric (M1) using RNA-Seq data. (XLSX)

S15 Table GO, KEGG, and BioCarta enrichment tests for BRCA modules constructed by hierarchical clustering with the PCC distance metric (M2) using RNA-Seq data. (XLSX)

S16 Table GO, KEGG, and BioCarta enrichment tests for BRCA modules constructed by K-means clustering with the PCC distance metric (M3) using RNA-Seq data. (XLSX)

S17 Table GO, KEGG, and BioCarta enrichment tests for BRCA modules constructed by CLICK clustering (M4) using RNA-Seq data. (XLSX)

S18 Table GO, KEGG, and BioCarta enrichment tests for BRCA modules constructed by SAMBA biclustering (M5) using RNA-Seq data. (XLSX)

S19 Table GO, KEGG, and BioCarta enrichment tests for BRCA modules constructed by NMF (M6) using RNA-Seq data. (XLSX)

S20 Table GO, KEGG, and BioCarta enrichment tests for integrated BRCA modules constructed by NMF (M7) using RNA-Seq data. (XLSX)

S21 Table GO, KEGG, and BioCarta enrichment tests for integrated BRCA modules constructed by NMF (M8) using RNA-Seq data. (XLSX)

S22 Table GO, KEGG, and BioCarta enrichment tests for BRCA modules constructed by FGMD (M9) using RNA-Seq data. (XLSX)

S23 Table TF enrichment test for BRCA modules using microarray data. (XLSX)

S24 Table TF enrichment test for BRCA modules using RNA-Seq data. (XLSX)

S25 Table BRCA gene enrichment test for BRCA modules using microarray data. (XLSX)

S26 Table BRCA gene enrichment test for BRCA modules using RNA-Seq data. (XLSX)

S27 Table Genes in OVC modules using gene expression data. (XLSX)

S28 Table Isoforms in OVC modules using isoform expression data. (XLSX)

S29 Table Performance comparison of OVC modules constructed by NMF in determining the the rank r. (XLSX)

S30 Table Comparison of enrichment tests for GO terms and BioCarta pathways for OVC modules. (XLSX)

S31 Table GO, KEGG, and BioCarta enrichment tests for OVC modules constructed by hierarchical clustering with the Euclidean distance metric (M1) using gene expression data. (XLSX)

S32 Table GO, KEGG, and BioCarta enrichment tests for OVC modules constructed by hierarchical clustering with the PCC distance metric (M2) using gene expression data. (XLSX)

S33 Table GO, KEGG, and BioCarta enrichment tests for OVC modules constructed by K-means clustering (M3) using gene expression data. (XLSX)

S34 Table GO, KEGG, and BioCarta enrichment tests for OVC modules constructed by CLICK biclustering (M4) using gene expression data. (XLSX)

S35 Table GO, KEGG, and BioCarta enrichment tests for OVC modules constructed by SAMBA biclustering (M5) using gene expression data. (XLSX)

S36 Table GO, KEGG, and BioCarta enrichment tests for OVC modules constructed by NMF (M6) using gene expression data. (XLSX)

S37 Table GO, KEGG, and BioCarta enrichment tests for OVC modules constructed by NMF (M7) using gene expression data. (XLSX)

S38 Table GO, KEGG, and BioCarta enrichment tests for OVC modules constructed by WGCNA (M8) using gene expression data. (XLSX)

S39 Table GO, KEGG, and BioCarta enrichment tests for OVC modules constructed by FGMD (M9) using gene expression data. (XLSX)

S40 Table GO, KEGG, and BioCarta enrichment tests for OVC modules constructed by hierarchical clustering with the Euclidean distance metric (M1) using isoform expression data. (XLSX)

S41 Table GO, KEGG, and BioCarta enrichment tests for OVC modules constructed by hierarchical clustering with the PCC distance metric (M2) using isoform expression data. (XLSX)

S42 Table GO, KEGG, and BioCarta enrichment tests for OVC modules constructed by k-means clustering (M3) using isoform expression data. (XLSX)

S43 Table GO, KEGG, and BioCarta enrichment tests for OVC modules constructed by CLICK clustering (M4) using isoform expression data. (XLSX)

S44 Table GO, KEGG, and BioCarta enrichment tests for OVC modules constructed by SAMBA biclustering (M5) using isoform expression data. (XLSX)

S45 Table GO, KEGG, and BioCarta enrichment tests for OVC modules constructed by NMF (M6) using isoform expression data. (XLSX)

S46 Table GO, KEGG, and BioCarta enrichment tests for OVC modules constructed by NMF (M7) using isoform expression data. (XLSX)

S47 Table GO, KEGG, and BioCarta enrichment tests for OVC modules constructed by WGCNA (M8) using isoform expression data. (XLSX)

S48 Table GO, KEGG, and BioCarta enrichment tests for OVC modules constructed by FGMD (M9) using isoform expression data. (XLSX)

S49 Table TF enrichment test for OVC modules using gene expression data. (XLSX)

S50 Table TF enrichment test for OVC modules using isoform expression data. (XLSX)

S51 Table Comparison of the performance rankings. (XLSX)

S52 Table Comparison of the execution time of all methods using BRCA gene expression data obtained from microarray. (XLSX)

S53 Table Performance comparison of modules depending on the parameter of ‘Dynamic TreeCuts’. (XLSX)

S54 Table Performance comparison of the FGMD algorithm with and without overlaps in small modules. (XLSX)

S1 Fig Comparison of the range of values between microarray and RNA-Seq. The distributions of expression values of microarray (A) and RNA-Seq (B) are shown. The *x*-axis represents expression values and the *y*-axis represents the number of points.
(TIF)

S2 Fig Comparison of the absolute PCC values for all gene pairs for microarray and RNA-Seq. (A) Distributions of the absolute PCC values of all gene pairs for microarray and RNA-Seq. (B) Distributions of the difference in absolute PCC values between microarray and RNA-Seq data for the same gene pairs.
(TIF)

S3 Fig Relationship between two platforms and comparison of the network statistics. (A) The distribution of PCC values between gene expression levels from microarray and RNA-Seq for the same genes (14,352 genes). The *x*-axis shows PCC values and the *y*-axis shows the number of genes. (B) Boxplots of total node connectivity and coefficient of variations.
(TIF)

S4 Fig Absolute PCC value differences between gene and isoform expression levels for the GGI pairs. (A) Distribution of PCC values for GGI pairs. Red and green lines represent the distributions of PCC values from gene and isoform expression levels, respectively. (B) Distribution of the absolute differences in PCC values between gene and isoform expression for the same gene pairs. (C) The top 20 GGI pairs showing large differences. In the table, “Microarray” and “RNA-Seq” represent PCC values for a gene pair using expression data from microarray and RNA-Seq, respectively, and “Difference” represents the difference in PCC values between the previous two values. “Rank” represents the ranking of the GGI pairs sorted according to the “Difference” values.
(TIF)

S1 File Comparison of gene expression of microarray and RNA-Seq for BRCA and comparison of gene and isoform expression of RNA-Seq for OVC. (PDF)
