# Supplementary figures and images for: FGMD: A novel approach for functional gene module detection in cancer
Source: PLoS One. 2017 Dec 15;12(12):e0188900. doi: 10.1371/journal.pone.0188900 (PMC5731741; doi:10.1371/journal.pone.0188900)

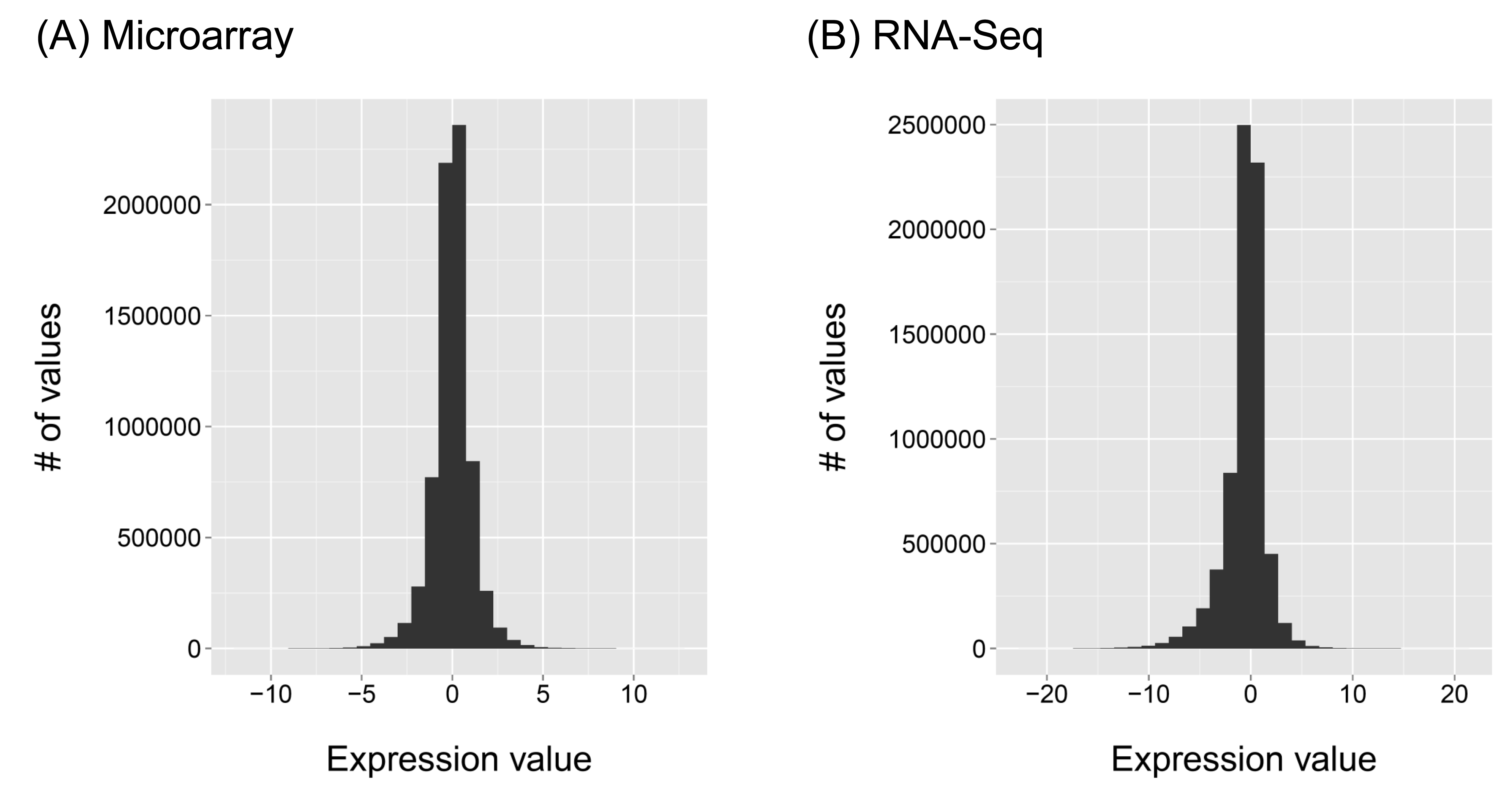

Supplement: S1 Fig — The distributions of expression values of microarray (A) and RNA-Seq (B) are shown. The x-axis represents expression values and the y-axis represents the number of points. (TIF) [file pone.0188900.s055.tif]

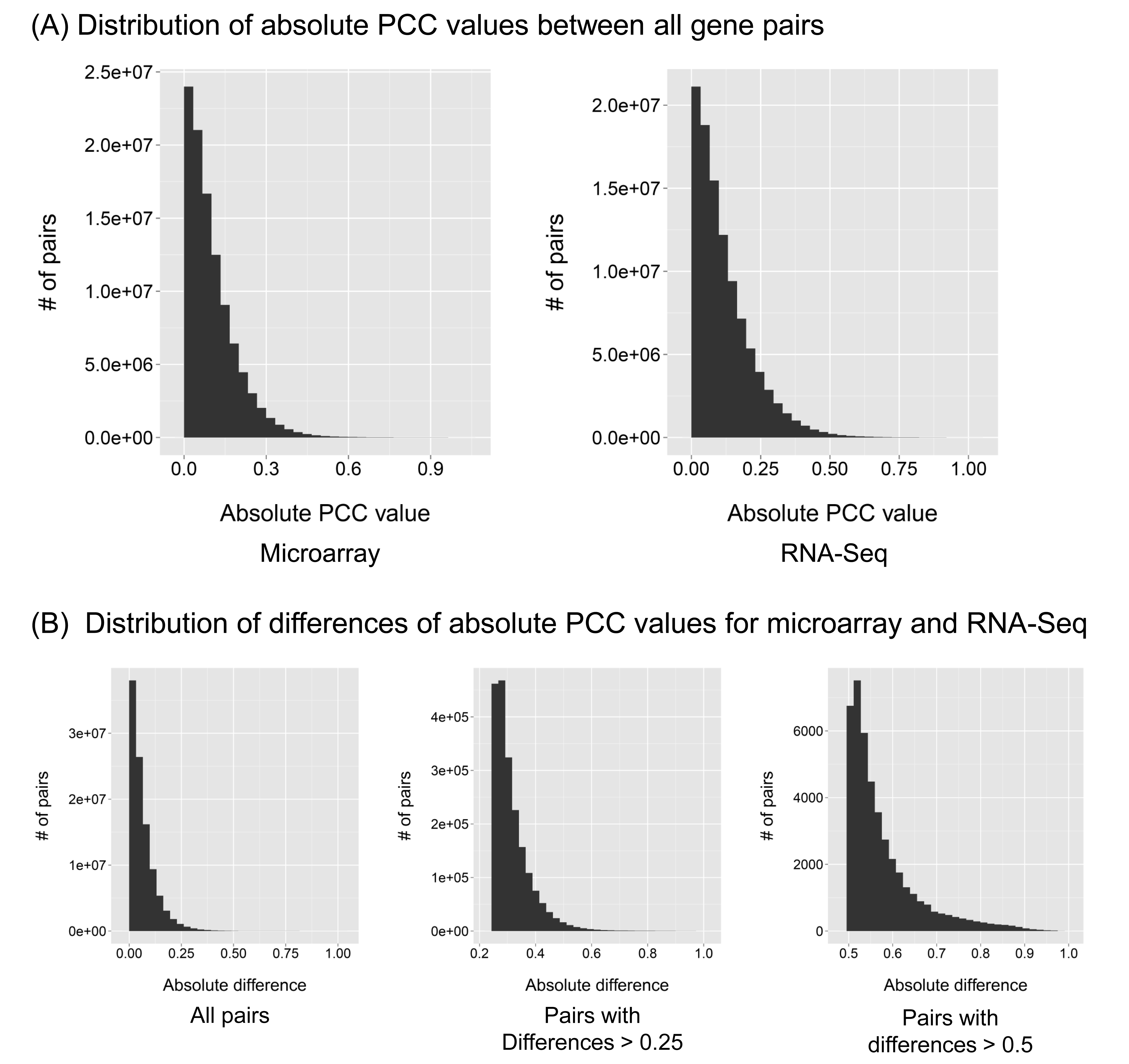

Supplement: S2 Fig — (A) Distributions of the absolute PCC values of all gene pairs for microarray and RNA-Seq. (B) Distributions of the difference in absolute PCC values between microarray and RNA-Seq data for the same gene pairs. (TIF) [file pone.0188900.s056.tif]

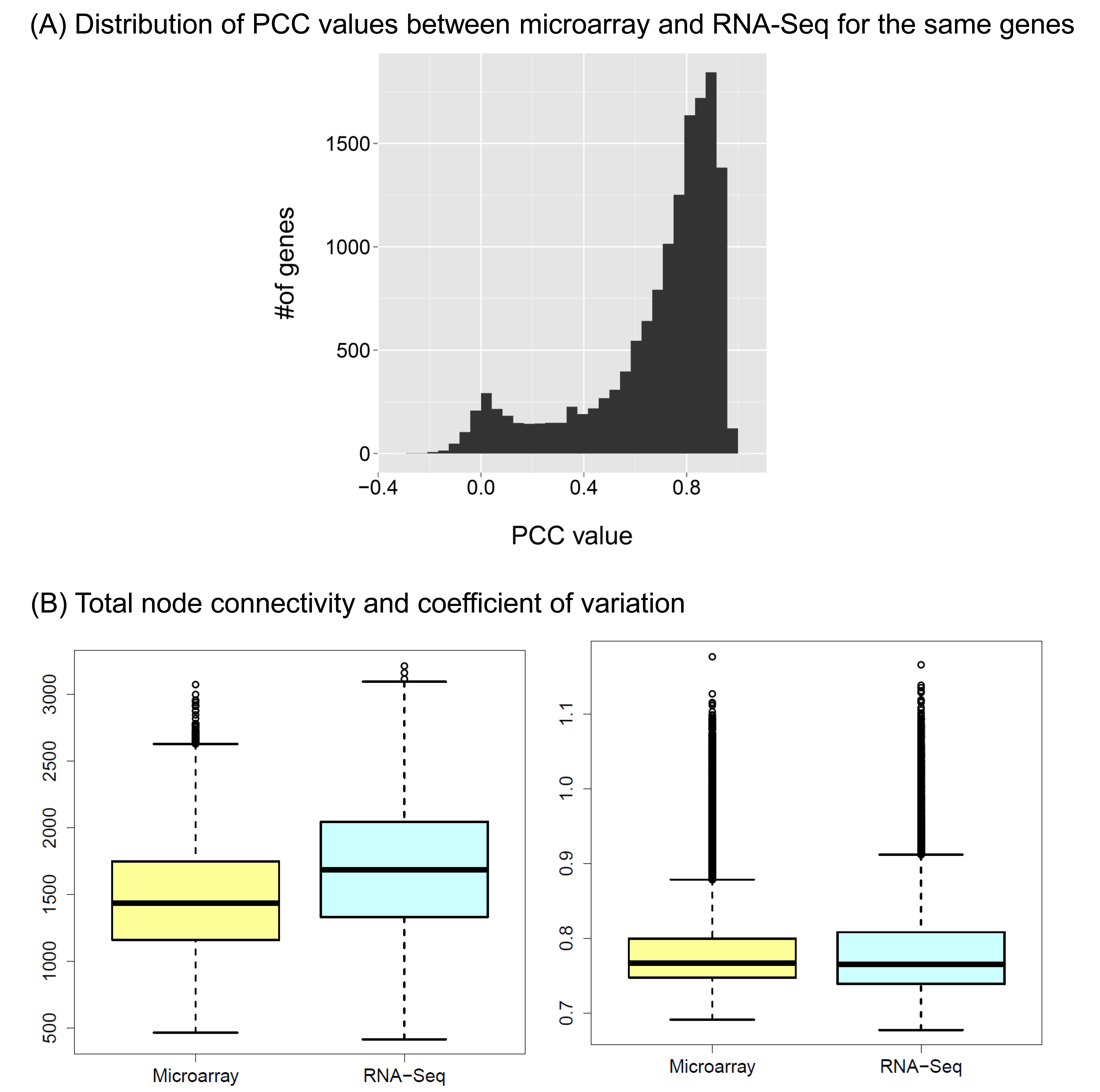

Supplement: S3 Fig — (A) The distribution of PCC values between gene expression levels from microarray and RNA-Seq for the same genes (14,352 genes). The x-axis shows PCC values and the y-axis shows the number of genes. (B) Boxplots of total node connectivity and coefficient of variations. (TIF) [file pone.0188900.s057.tif]

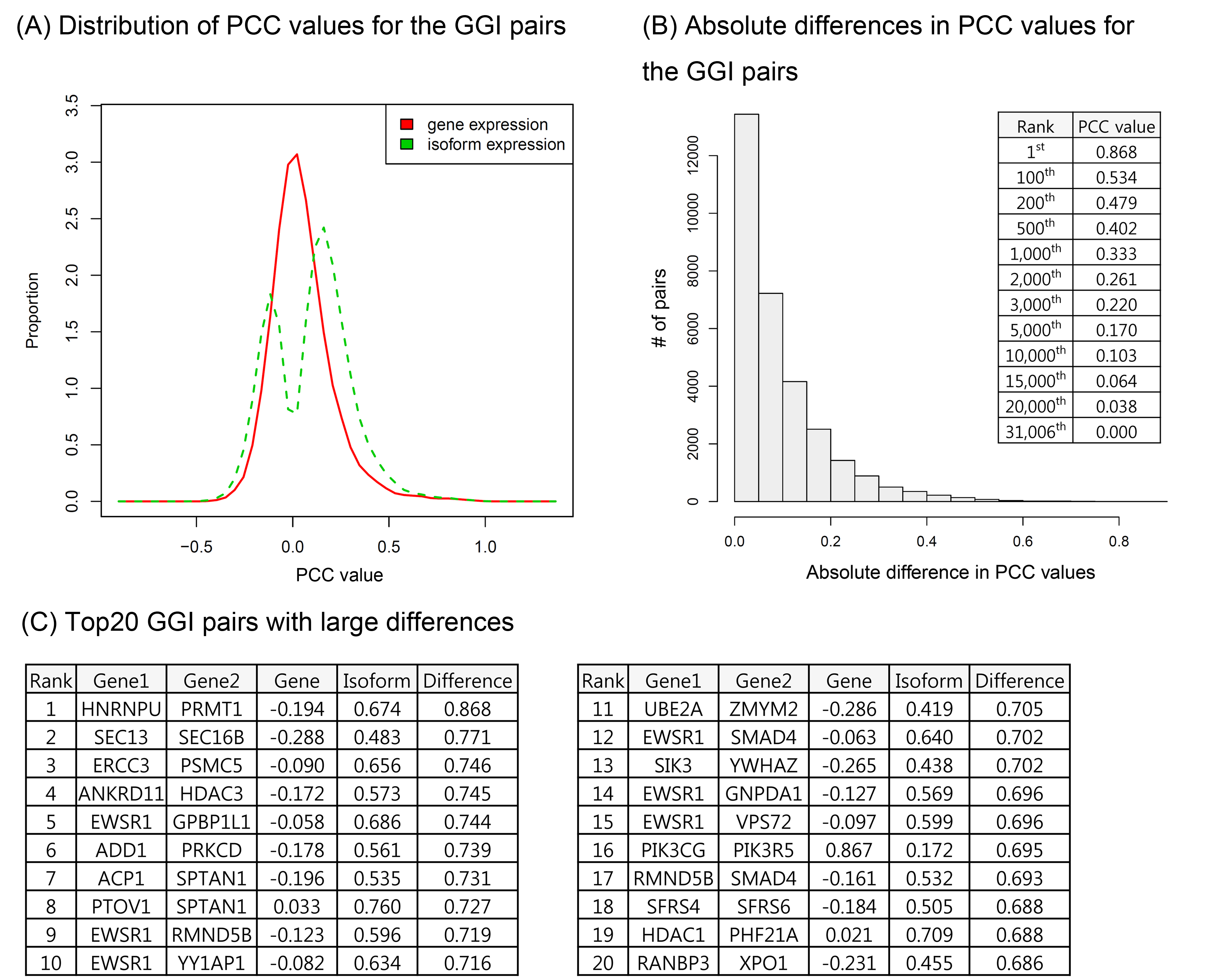

Supplement: S4 Fig — (A) Distribution of PCC values for GGI pairs. Red and green lines represent the distributions of PCC values from gene and isoform expression levels, respectively. (B) Distribution of the absolute differences in PCC values between gene and isoform expression for the same gene pairs. (C) The top 20 GGI pairs showing large differences. In the table, “Microarray” and “RNA-Seq” represent PCC values for a gene pair using expression data from microarray and RNA-Seq, respectively, and “Difference” represents the difference in PCC values between the previous two values. “Rank” represents the ranking of the GGI pairs sorted according to the “Difference” values. (TIF) [file pone.0188900.s058.tif]
