# Supplementary material for: FGMD: A novel approach for functional gene module detection in cancer
Source: PLoS One. 2017 Dec 15;12(12):e0188900. doi: 10.1371/journal.pone.0188900 (PMC5731741; doi:10.1371/journal.pone.0188900)
Supplement: S1 File — (PDF) [file pone.0188900.s059.pdf]

Supplementary Material for FGMD: A Novel Approach for Functional Gene Module Detection in Cancer

Daeyong Jin[1] and Hyunju Lee[1*]

1 School of Electrical Engineering and Computer Science, Gwangju Institute of Science and Technology, Gwangju, South Korea

* hyunjulee@gist.ac.kr

Comparison of Gene Expression of Microarray and RNA-Seq for BRCA

We compared the expression profiles of 14,352 genes from microarray and RNA-Seq datasets. We first checked the range of expression values for 7,061,184 points from 14,352 genes for 492 samples. Figure S1 shows the distribution of gene expression values. For the microarray data, the maximum, minimum, and average values were 11.468, -11.118, and -0.007, respectively. For the RNA-Seq data, the maximum, minimum, and average values were 18.986, -21.204, and -0.556, respectively. Thus, the expression values from RNA-Seq showed a broader range than those from the microarray.

Second, for 14,352 genes, we computed the absolute PCC values among all gene pairs from the microarray and RNA-Seq datasets. Figure S2 shows the distribution of absolute PCC values among all gene pairs, including 102,982,776 points (14,352 $\times$ 14,351/2). For microarray, the maximum, minimum, and average values were 0.997, 5.03e-09, and 0.101, respectively. For RNA-Seq, the maximum, minimum, and average values were 0.986, 5.13e-09, and 0.117, respectively. When comparing the absolute PCC values for gene pairs using a a pairwise t-test, RNA-Seq showed higher PCC values than microarray with a p-value $< 2.2e\text{-}16$. In addition, we computed the difference in absolute PCC values between microarray and RNA-Seq for the same gene pairs, and the distribution of the difference in absolute PCC values is shown in Fig. S2 (B). The average of the difference was 0.06, implying that most of the relationships between genes was similarly represented in the two platforms. However, some gene pairs showed a significant difference (e.g., 43,339 pairs had a difference in PCC values > 0.5), implying that the relationship between some genes might only be detected in one platform.

Figure S3 shows the distribution of PCC values for the same genes from microarray and RNA-Seq. The average value was 0.683, demonstrating the similar tendency of expression changes in the two platforms. Fig. S3 (B) shows boxplots of the connectivity and coefficient of variation, and Table below shows the global network statistics of density, centralization, and heterogeneity. Density represents the average of absolute PCC values, indicating the average strength of relationships among genes. High centralization represents the difference between density and the total connectivity value of a gene with a maximum value. Heterogeneity indicates the variability in total node connectivity values. A previous study [1] that compared gene expression data between RNA-Seq and microarray showed that correlations among gene expression values measured using RNA-Seq constitute a more densely connected gene network with higher density, centralization, and heterogeneity than those derived from the microarray. Similarly, our results showed that a gene network from expression values measured using RNA-Seq has higher density and variability, confirming the findings of the previous

study, although the gene network from the microarray showed slightly higher centralization than that from RNA-Seq.

Table. Global network statistics for BRCA.

	Microarray	RNA-Seq
Density	0.102	0.117
Centralization	0.112	0.107
Heterogeneity	0.297	0.341

Comparison of Gene and Isoform Expression of RNA-Seq for OVC

We first compared the gene and isoform expression profiles for OVC data. We checked the range of expression values for 5,974,521 and 21,417,309 points from 20,531 genes and 73,599 isoforms in 291 samples. For the genes and isoforms, we computed the average expression values and then computed the minimum, 25th percentile, median, 75th percentile, and maximum values, which were 0, 19.003, 264.674, 922.368, and 115532.413 for gene expression, and 0, 1.613, 18.34, 138.795, and 431348.301 for isoform expression, respectively. Considering the data distribution, we filtered 25% and 50% of the lowly expressed genes and isoforms, respectively, based on average expression values that were close to zero, and additionally filtered genes or isoforms whose values were zero for more than half of the samples. After filtering, we used 15,374 genes and 35,535 isoforms for the construction of FGMD modules.

When we checked the distribution of PCC values, for gene expression, the maximum, minimum, and average values were 0.997, 5.03e-09, and 0.101, respectively. For isoform expression, the maximum, minimum, and average values were 0.986, 5.13e-09, and 0.117.

Figure S4 shows the distribution of PCC values from gene and isoform expression data for validated gene-gene interaction (GGI) pairs from the HPRD [2], showing that the PCC values of isoform expression were slightly higher than those of gene expression. Fig. S4 (B) shows the distribution of the difference in the absolute PCC values for the gene pairs in the GGI using gene and isoform expression data. Of the 31,006 GGI pairs, 1,046 showed large differences in PCC values (>0.3). Fig. S4 (C) shows the top 20 GGI pairs with the largest differences in PCC values. This result demonstrates that although many studies combine a set of isoform expression data for a single gene, using the isoform expression itself can help to construct different functional gene modules.

References

1. Iancu OD, Kawane S, Bottomly D, Searles R, Hitzemann R, McWeeney S. Utilizing RNA-Seq data for de novo coexpression network inference. Bioinformatics. 2012;28(12):1592–1597.

2. Prasad TK, Goel R, Kandasamy K, Keerthikumar S, Kumar S, Mathivanan S, et al. Human protein reference database?2009 update. Nucleic acids research. 2009;37(suppl 1):D767–D772.
